# Research into the Association of Cadmium and Manganese Excretion with Thyroid Function and Behavioral Areas in Adolescents with Autism Spectrum Disorders

**DOI:** 10.3390/jcm11030579

**Published:** 2022-01-24

**Authors:** Anna Błażewicz, Ewelina Grywalska, Paweł Macek, Paulina Mertowska, Sebastian Mertowski, Julia Wojnicka, Nicolo Durante, Agata Makarewicz

**Affiliations:** 1 Department of Pathobiochemistry and Interdisciplinary Applications of Ion Chromatography, Medical University of Lublin, 1 Chodźki Street, 20-093 Lublin, Poland; julia.wojnicka@umlub.pl (J.W.); a.blazewicz@umlub.pl (N.D.); 2 Department of Experimental Immunology, Medical University of Lublin, 4a Chodźki Street, 20-093 Lublin, Poland; ewelina.grywalska@umlub.pl (E.G.); paulinamertowska@gmail.com (P.M.); mertowskisebastian@gmail.com (S.M.); 3 Department of Oncology, Institute of Health Sciences, Collegium Medicum, Jan Kochanowski University, 25-713 Kielce, Poland; pawel.macek@gazeta.pl; 4 Department of Epidemiology and Cancer Control, Holycross Cancer Centre, 25-734 Kielce, Poland; 5 Department of Psychiatry, Psychotherapy and Early Intervention, Medical University of Lublin, 20-439 Lublin, Poland; chemistry_coordinator@umlub.pl

**Keywords:** thyroid-stimulating hormone, urinary cadmium, urinary manganese, autism spectrum disorders, body mass index

## Abstract

Thyroid dysfunction and toxic metal exposure have been linked to the increased risk of autism spectrum disorders (ASD); however, the relationship between those factors remains unclear. We aimed to evaluate the relationship between the serum level of hormones, namely thyroid-stimulating hormone (TSH), free triiodothyronine (fT3), free thyroxine (fT4), and urinary cadmium (U-Cd) and urinary manganese (U-Mn), in patients with ASD. The study group consisted of 129 adolescents with ASD, and the control group consisted of 86 healthy persons. Ion chromatography with spectrophotometric detection (IC-UV/ViS) was used to quantitatively determine Cd and Mn in all 24-h urine samples. These results indicate that severity of certain symptoms in autism is associated with thyroid function. Correlation analysis between Childhood Autism Rating Scale (CARS) results and the content of both U-Mn and U-Cd as well as fT3, fT4 and TSH values in ASD patients showed significantly positive correlation of CARS7 (visual reaction) with fT3 and fT4 and a negative correlation with TSH for the whole study group. In the group of adolescents over 14 years of age, it was also observed that CARS10 (anxiety reaction) negatively correlates with serum TSH levels, and among younger individuals, CARS9 (near receptor responsiveness, taste, smell) positively correlates with TSH.

## 1. Introduction

Autism spectrum disorders (ASD) is a group of complex, heterogeneous, neurodevelopmental conditions. The International Statistical Classification of Diseases and Related Health Problems (the 10th revision, ICD-10) list by the World Health Organization (WHO) distinguishes a group of pervasive developmental disorders characterized by qualitative abnormalities in reciprocal social interactions and in patterns of communication and by a restricted, stereotyped, repetitive repertoire of interests and activities. That group includes childhood autism; atypical autism; Rett syndrome, another childhood disintegrative disorder; hyperkinetic disorder with associated mental retardation and stereotyped movements; Asperger’s syndrome, another global development disorder; and comprehensive developmental disorder, unspecified. Common features of all these disorders are problems in communication and understanding of social phenomena [1].

**Figure 1:**
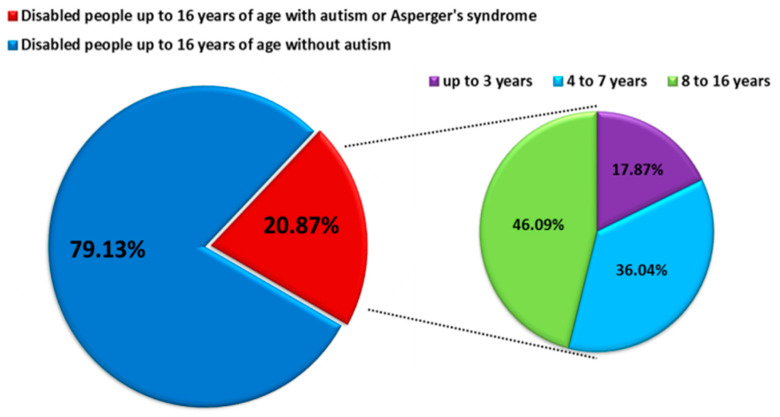
Number of persons with diagnoses of comprehensive developmental disorders in 2010–2019 in Poland (modified by data from https://www.nik.gov.pl/aktualnosci/wsparcie-osob-z-autyzmem-i-zespolem-aspergera.html, accessed on 29 September 2021).

ASD is characterized by impairment of social behavior, social interactions, communication, learning, and the presence of restricted and repetitive patterns of behavior and activities [2,3]. ASD affects more children than cancer, diabetes, and AIDS combined, which makes it the fastest-growing neurodevelopmental disability, with a higher prevalence among males than females [4,5]. According to literature data, the prevalence of ASD in the world is about 1 percent of the entire population of individual countries. For 2017, the prevalence was, respectively, 0.74% for the United States, 0.94% for Canada, 0.64% for Germany and Australia, and 0.47% for Poland. Detailed analyses based on the DALY (Disability Adjusted Years of Life) for ASD measured per 100,000 people showed that this rate is highest for Canada at 129.54 and then the United States at 101.57, while for European countries, it ranges from limits 93–62 DALY’s per 100,000 people. The DALY index is used to measure the overall burden of disease (both years lost and years lived with disability), and 1 DALY is understood as one healthy life year lost [6]. Based on the data from the Centers for Disease Control and Prevention (CDC), the prevalence of ASD increases year by year: at the beginning of 2000, it was 1 in 150 people over eight years, while in 2016, it was 1 in 54 people [7]. Similar trends are observed in many European countries, including Poland, while in the years 2010–2019, the percentage of disabled children diagnosed with ASD was 20.87%, and detailed data on age showed that the largest percentage was constituted by school children and adolescents 8–16 years old (46.09%) (Figure 1) [8]. Because ASD is a lifelong condition, the management of ASD is associated with huge health-economic costs [5,9] The lifetime social cost for an individual with an ASD in the U.S. was estimated at $3.6 million. For all of the individuals with ASD identified in the three decades from 1990–2019, the lifetime social cost for the U.S. is estimated to be more than $7 trillion in 2019 dollars [10].

Apart from being heterogeneous in nature, ASD is believed to be multifactorial in origin. However, the pathogenesis of ASD remains poorly understood, and there is no consensus among investigators regarding the exact etiology. It is suggested that genetic, biological, and environmental factors altogether contribute to the development of ASD. ASD is known to be highly heritable (~90%), and some related genes have been reported [11,12,13]. Despite that, a genetic component cannot fully explain ASD as a single gene, and chromosomal defects only account for a minority of ASD cases (10–20%) [14,15]. More and more studies raise the importance of environmental factors in the development of ASD. This applies to contamination and/or exposure of the developing organism to many metals, such as lead, aluminum, or cadmium, as well as to disturbances in the homeostasis of microelements, such as manganese or zinc, the presence of which is necessary for the proper functioning of the organism [16]. Manganese is one of the most common metals found on Earth and thus is a source of metal used in many industrial products, including leather, textile, battery production, glass, as well as cosmetics, paints, and fertilizers. This means that it is present in almost every sphere of human life, constituting not only a micronutrient necessary for proper functioning but also a potential source of danger. Estimated literature data indicate that the content of Mn in soils in the United States varies from 40 to 900 mg/kg, which directly affects its presence in plants (500 µg/g is toxic to plants) as well as in the meat of farm animals that we eat. There are several ways for Mn to enter the human body, including alimentary (diet, water, food), inhalation (air), or skin contact [17,18,19]. Although an exposure to excess levels of manganese has many negative effects, including neurological disorders (tremors, difficulty walking, muscle and facial spasms), impaired cognitive effects (problems with concentration, memory, decreased scientific performance), and neurobehavioral disorders (hyperactivity, increased behavior opposition), it should not be fully eradicated from the diet, as it also has many health-promoting functions [16,17,20]. Such functions include the protection of brain cells against oxidative stress or the influence on the release of neurotransmitters. It should also be mentioned that Mn deficiency in the body can lead to growth impairment, poor bone formation, and skeletal defects as well as reduced fertility, birth defects in children, and metabolic changes (impaired glucose tolerance, reduced lipid and carbohydrate metabolism) [16,17,18,19,20]. The homeostasis of Mn ions in the body is maintained and regulated by a number of mechanisms of uptake, storage, and release by individual cells. About 3–5% of ingested Mn is absorbed from the gastrointestinal tract. It is thought that under homeostatic conditions, Mn enters the portal circulation through either passive diffusion or active transport involving divalent metal transporter 1 (DMT1). Mn is distributed throughout the body, including bones, kidneys, pancreas, liver, and brain, which accumulate the highest contents of Mn [21].

Inhaled Mn is very often transported directly to the brain before it is metabolized by the liver. This is clearly visible in the MRI pictures, where the excess of this element is accumulated in the CNS in basal ganglia, which in humans plays important functions in the processes of body movement control, cognitive processes, emotional control, and learning [18,19].

In addition to Mn, the second most significant metal influencing the functioning of the human body is cadmium, belonging to mutagenic and teratogenic elements. Like other heavy metals, Cd is present in the environment as a result of many anthropogenic activities, including use in industry as a corrosive reagent, as a PVC stabilizer, in batteries, in accumulators, from smelting and refining copper and nickel, from burning fossil fuels, and even from the use of fertilizers phosphate. Due to the ubiquitous presence of this metal in the environment, there are three ways for Cd to enter the body: mainly through the respiratory tract, through the gastrointestinal tract, and less often through the skin [22]. Upon entering the body, Cd is transported by erythrocytes and albumin and accumulates in the kidneys, liver, and intestines. The presence of this element in the body causes a number of adverse health effects, such as kidney and adrenal dysfunction, liver disorders, pulmonary edema, damage to the genital organs, and disorders of the hematopoietic system. Cd is also a common carcinogen involved in the development of breast, lung, prostate, bladder, nasopharyngeal, and pancreatic cancers [22,23].

Thyroid hormones are essential for the proper functioning of the human body due to the maintenance of balance in almost all metabolic processes. Triiodothyronine (T3) and thyroxine (T4) in the early stages of development ensure normal growth as well as the functioning of the nervous and cardiovascular systems. Their level varies depending on many factors, e.g., in elderly people, it drops from 10 to 50% compared to children, but their impact on many metabolic processes and circulation, functioning of the digestive tract, muscles, nerves, and mental functions remains unchanged [24]. The thyroid-stimulating hormone (TSH) produced by the pituitary gland in order to stimulate the secretion of T3 and T4 by the thyroid gland is also involved in the proper functioning of the thyroid gland. The thyroid gland is often exposed to many endocrine toxic elements, such as cadmium or manganese, causing disorders of proper functioning [18]. Mn ions can directly and indirectly interfere with the binding, transport, and activity of thyroid hormones at the tissue level. The first pathway concerns the dysfunction of deiodinase enzymes (which are responsible for the activation and deactivation of thyroid hormones) involved in the metabolic, signaling, and regulatory processes of the formation and conversion of thyroid hormones, whereas the second pathway concerns the binding of Mn ions to dopaminergic receptors. Dopamine exerts a suppressive effect on TSH secretion and thereby affects the level of thyroid hormones (damage to dopaminergic receptors may cause non-developmental deficits) [18,19].

In the case of Cd, literature data indicate structural and functional damage to the follicle and the endocrine apparatus of the thyroid gland. This is indicated by histological changes (flattening of follicular cells, growth of interstitial fibrous tissue between the follicles) and metabolic changes in the functioning of the thyroid gland. A positive correlation has also been demonstrated between the concentration of Cd in the blood and its accumulation in the thyroid gland. It was reported that the thyroid gland of people exposed to cadmium accumulates three times more cadmium compared to that of unexposed people [25]. Studies in an animal model have shown that Cd reduces the secretory granules of thyroglobulin in the cytoplasm, which also causes a decrease in serum T3 and T4 levels [22,23,26].

It has been shown that thyroid dysfunction is associated with neurological and psychiatric disorders, including neurodevelopmental disorders, such as ASD [27,28,29,30,31,32]. However, the exact relationship is far from being fully understood. It has been shown that a family history of autoimmune thyroid disease was associated with ASD with developmental regression [33], and it was suggested that maternal hypothyroidism is associated with ASD in children [34]. Another study showed no differences between thyroid hormone levels measured in children already diagnosed with ASD compared with typically developing controls [35]. One study found that very low thyroxine (T4) levels (3rd percentile) in children were associated with higher ASD risk [31], while the other study found no association between neonatal T4 levels and ASD or any other neurological condition [36]. Furthermore, several toxic metals, including arsenic (As), lead (Pb), aluminum (Al), cadmium (Cd), and mercury (Hg), which are widely present in the environment, have been classified as neurotoxins [37,38,39]. Yasuda et al. [40] indicated that Cd highly accumulates in infants and children. Later, the same authors demonstrated high burdens of Cd in infantile patients diagnosed with autism [41].

Rossignol et al. [42] reported in a meta-analysis on toxicants and ASD that almost three-quarters of research found significant differences between neurotypical individuals and individuals with ASD. On the other hand, some studies revealed that children with ASD are characterized by lower values of toxic elements (e.g., As and Pb) in hair [43].

Both effects of thyroid dysfunction [32,44] and exposure to toxic metals [45] in ASD have already been discussed in literature. Our study aimed to assess the relationship between the level of hormones influencing the proper functioning of the thyroid in the serum: thyroid-stimulating hormone (TSH), free triiodothyronine (fT3), free thyroxine (fT4), and the concentration of cadmium (U-Cd) and manganese (U-Mn)) in urine of patients with ASD. We hoped that our research results would allow us to enrich the literature data and allow us to shed new light on the process of ASD pathogenesis in adolescents.

In the light of the existing inconsistencies regarding study designs and findings, and the lack of precisely known mechanisms underlying the effects of heavy metals as endocrine disruptors conducting this type of research is justified.

## 2. Materials and Methods

### 2.1. Study Design

The study protocol underwent review and approval by the Ethics Committee of the Medical University of Lublin, Poland (KE-0254/12/2014, and KE-0254/12/2016).

The participants reported in this study were part of a larger investigation project initiated by Anna Błażewicz at the Medical University of Lublin entitled “Application of IC and ICP-MS techniques to study the correlation between levels of trace elements and severity of symptoms of autism” (KE-0254/12/2014) and “Study of the influence of environmental factors on the occurrence and character of autism spectrum disorders” (KE-0254/12/2016). The studies were performed following The Code of Ethics of the World Medical Association (Declaration of Helsinki) for experiments involving humans. The parents or guardians of the studied subjects provided informed and written consent. Patients were recruited for the research in cooperation with local support groups or by referral by specialist doctors and therapists. The diagnostic process of ASD was conducted by a multidisciplinary team (including physicians, psychologists, and therapists). The final diagnosis of autism spectrum disorder was made by a specialist in psychiatry. Standardized diagnostic tools, such as the Autism Diagnostic Observation Schedule, Second Edition (ADOS-2) [46], and the Autism Diagnostic Interview—Revised (ADI-R) [47], allowed to gather comprehensive information about the development of the diagnosed person. On their basis, it was possible to assess the observed abnormal characteristics of autism spectrum disorders manifesting themselves in four key spheres: social, communication, behavior and interests, and imagination and creativity.

The classified patients fulfilled the diagnostic criteria of autism set out in ICD-10 [1] and the 5th edition of the Diagnostic and Statistical Manual of Mental Disorders American Psychiatric Association [2]. In the case of patients recruited by local support groups, parents or guardians of patients presented medical certificates confirming the diagnosis of ASD. Verification of diagnosis by MUL staff was based on the Autism Diagnostic Observation Schedule (ADOS-2) and Childhood Autism Rating Scale (CARS-2).

The criteria for including patients in the study group were as follows: age 12–17 years, diagnosis of ASD, no supplementation with minerals and vitamins at least 3 months before sampling, and no thyroid pathology or treatment related to endocrine or infectious diseases. On this basis, 129 patients aged 12–17 years (108 male and 21 female) were included in the study. The control group consisted of 86 neurotypical people aged 12–17 years, including 32 female and 54 male. Patients were recruited to the control group from among children coming to the clinic for regular check-ups. The lack of diagnosis of ASD in the control group was based on the explanations of the parents or guardians that they did not find any symptoms in their charges allowing for the diagnosis of ASD and the therapists’ check-ups. The criteria for including patients in the control group were as follows: age 12–17, no diagnosis of ASD, no supplementation with minerals and vitamins at least 3 months before sampling, and no thyroid pathology or treatment related to endocrine or infectious diseases. At the time, they were not receiving any medical treatment and were not admitted to the hospital. These participants were unrelated to the autistic patients. The socioeconomic status of all participants was similar, and all adolescents were recruited from the same area, namely the southeast, the unindustrialized region surrounding Lublin, Poland—a city of approximately 400,000 residents.

Research on the autism severity scale and behavioral rating scale (i.e., The Childhood Autism Rating Scale (CARS -2) [48] has been described in detail in one of our previous papers [49]. The questionnaire rates the child on a four-point scale in each of fifteen criteria (scoring standards are as follows:1—within the normal range for child’s age, 2—mildly abnormal, 3—moderately abnormal, 4—severely abnormal). Detailed data on the mean, standard deviation, median, and range level of response of ASD patients in individual CARS are presented in Table 1.

### 2.2. Sample Collection and Measurements

An initial personal interview was conducted by trained personnel to establish individual’s health status. A child’s weight status was determined using an age- and sex-specific percentile for body mass index (BMI, in kg/m^2^). Current and representative BMI percentile charts for the Polish population of children and adolescents (3–18 year of age) were used [49]. Measurements of serum-free triiodothyronine (fT3), free thyroxine (fT4), and thyroid-stimulating hormone (TSH) were performed by accredited diagnostic labs (Lublin, Poland) holding certificates according to PN-EN ISO 9001:2009 standards. A fasting blood from the cephalic vein was collected in the morning and processed immediately. Since the non-invasive sampling from our young patients was a priority, we collected 24-h urine samples for Cd and Mn quantitative measurements. Excretion of elements was expressed in mass/day (24 h). Adjustment of concentrations for creatinine or specific gravity was not necessary since variability in urinary flow rate is less relevant in 24-h urine samples.

Samples taken from all the groups were pre-treated and analyzed in the same way. Samples were collected in disposable sterile plastic urine containers and stored at −25 °C before the sample pre-treatment procedure, i.e., microwave-assisted digestion with the use of 10 mL of digestion mixture (composed of 3 mL of 69.0% nitric acid (HNO_3_) solution and 7 mL of deionized water (resistivity 18.2 MΩ/cm)) (NovaWAVE Microwave Tunnel Digestion System (SCP Science, Baie-d’Urfé, Quebec, QC, Canada). The average recovery amounted to 98.2%. The optimized time of the digestion procedure did not exceed 30 min, including the cooling stage. The temperature was set at 180 °C. Reagents for the sample preparation procedure were of Suprapur^®^ grade (Merck, Darmstadt, Germany).

Ion chromatography with spectrophotometric detection (IC-UV/ViS) was used to determine quantitatively cadmium and manganese ions in all urine samples. IC serves as a fast, accurate, and precise method used for the determination of ions in urine and in other biological fluids at low µg/L levels [50,51]. The detailed operating parameters of the IC measurements can be found in the above-mentioned paper [51].

### 2.3. Statistical Analyses

Basic statistics are presented as mean ± standard deviation and median with minimum and maximum values. Statistical differences in the autism and control groups were assessed by *t*-test (equal variance), Welch test (unequal variance), or chi-square test (categorical variables). Equality of variance was checked with the F-test. The distribution of the analyzed variables were examined by the Shapiro−Wilk test. A *p*-value < 0.05 was considered significant. The relationships of two random variables were investigated using non-parametric Kendall’s tau correlation. The strength and direction of the correlation were presented using the correlation coefficient ranging from −1 to 1. The *p*-values and confidence intervals for the tested elements were replaced by Bayes Factor (BF) and 95% credible interval. The Beta prior width = 1, assigning equal probability to all correlation values between −1 and 1. BF_10_ presented in the tables and figures meant the Bayes Factor in favor of H1 (two-sided, non-directional alternative hypothesis, i.e., a relationship between variables) over H0 (null hypothesis, i.e., no relationship between variables). BF_10_ = 1 meant that both hypotheses predicted the data equally well. BF_10_ > 10 meant more support for H1 than H0. Since Bayes Factors are sensitive to the prior distribution method, a robustness analysis was performed. Robustness analysis calculates the BF10 values for all prior shape parameters between 0 and 2, indicating to what extent the BF changes based on the prior specification. All analyses were performed in JASP (Version 0.14.1) (Computer software). All analyses were performed based on the division of study participants by age (participants aged under 14 and participants 14 and over years of age) and gender.

## 3. Results

### 3.1. Basic Characteristics of Study Group

The study included 215 adolescents aged 12–17 years (mean age 14.3 years). The study group consisted of 129 adolescents with ASD (83.7% male and 16.3% female), while the control group consisted of 86 healthy persons (62.8% male and 37.2% female). The examined patients by a specialized psychiatrist, and healthy people were additionally divided into subgroups according to their sex and age categories: participants aged under 14 and participants 14 and over years of age. For all subgroups and the entire group, the concentrations of selected biochemical parameters reflecting the functioning of the thyroid gland were determined, i.e., the concentrations of TSH, fT3, and FT4 in the serum and the fT3/fT4 ratio, which reflects the T4 to T3 conversions through the enzymes of deiodinases, which is a sign of the current condition of the thyroid gland. The concentration of Mn and Cd ions in the urine of the examined persons was also determined as well as the BMI index. The average values of the analyzed parameters as well as their medians and ranges of occurrence of values, taking into account statistical significance, are presented in Table 2.

Taking into account all participants with ASD, it was shown that mean values of the body mass index (BMI), TSH, U-Mn, and U-Cd were significantly higher in the ASD group compared to the control group (Table 1). There were no significant differences in the mean levels of fT3, fT4, and fT3/fT4 in the studied groups. The analysis of individual subgroups showed that in the case of age differences in both examined categories, there are statistically significant higher mean values for the BMI index and U-Mn and U-Cd in patients with ASD compared to the control group. The TSH level is higher among ASD patients aged 14≥ and among males compared to the control group. Additionally, the fT4 level is significantly higher among ASD patients under the age of 14 and among females than in the control group. In the case of males and females with ASD, the BMI, U-Mn, and U-Cd indices were significantly higher.

In assessing the determinants of thyroid function, the large heterogeneity in the pediatric reference ranges should be taken into account. Published reference ranges for TSH, fT3, and fT4 vary by age and within age ranges, e.g., for children 6–11 years of age, the norms for fT4 are 12.5–21.5 pmol/L and for fT3 are 3.88–8.02 pmol/L, while at the age of 11–20, they are 12.6–21 pmol/L and 3.93–7.70 pmol/L, respectively [52]. The literature data show that a child’s age, sex, ethnicity, anthropometric data, and also time of venipuncture are determinants of TSH and/or fT4 concentrations [53]. In Poland, the reference ranges 0.4–5.0 mIU/L for TSH and 8–17 pmol/L for fT4 at the age of 14 y proposed by Fisher et al. are often adopted; for adolescents aged 14 years, they are fT4 8–17 pmol/L and for TSH 0.4–5.0 mIU/L [54]. Regardless of age, thyroid hormones affect many cellular processes, the proper functioning of the human body, and its mental functions; therefore, it seemed extremely important to examine thyroid function in patients with ASD also in the context of the potential disturbed elemental homeostasis.

### 3.2. Analysis of the Correlation between the Tested Biochemical Parameters Influencing the Functioning of the Thyroid Gland and the Concentration of U-Mn and U-Cd and the BMI Index in Patients with ASD and the Control Group

The conducted analyses showed that within the entire research group in individuals with ASD, there was a statistically significant positive correlation between the BMI values and the concentration of U-Cd, TSH, and the fT3/fT4 ratio. This means that the mean amounts of Cd in urine, serum TSH concentration, and the fT3/fT4 ratio also increased with the increase in BMI (Table 3).

Additionally, statistically significant changes in fT3 and fT4 concentrations with BMI values were observed. As the BMI value increased, the mean serum concentrations of fT3 and fT4 decreased, which is indicated by negative correlations. Detailed analysis of the examined subgroups of patients with ASD showed significant dependencies of BMI values and biochemical parameters depending on the age and sex of the examined people. By analyzing the age of the patients, statistically significant relationships were found between the increase in BMI value and the increase in the amount of U-Cd and serum TSH concentration (positive correlation) and a decrease in fT3 and fT4 levels (negative correlation) in group age ≥ 14. Among patients over 14 years of age, a statistically significant positive correlation was only between BMI and TSH and a negative correlation between BMI and fT4 (Table 2). There were also observed statistically significant relationships between the examined parameters and BMI in the context of sex differences. Both males and females showed an increase in mean serum TSH concentrations, which was positively correlated with BMI values. In addition, in the male group, significant relationships were also found between the BMI index and the concentration of U-Cd and fT3/fT4 (positive correlation), while a negative correlation was shown with the levels of fT3 and fT4 in the serum. Moreover, no statistically significant differences between the patients’ BMI and U-Mn values were found in any of the analyzed cases (Table 2). The only statistically significant correlation among the control group was observed in the case of TSH and the BMI (positive correlation) in the female group. However, the values obtained are 31.77% lower than those observed for females with ASD.

### 3.3. Analysis of the Correlation between U-Mn and U-Cd and the Biochemical Parameters of Thyroid Functioning in Patients with ASD and in the Control Group, Depending on the Age and Sex of the Examined Participants

As a result of the application of the two-sided alternative hypothesis (H1 ≠ H0) for the entire study group, it was found that with the increase in fT3 concentration, the U-Cd value decreased (negative correlation) in patients with ASD. Additionally, the same relationship was observed for fT4 and U-Mn and U-Cd. A positive correlation was also found for TSH and U-Mn as well as TSH and U-Cd in the ASD group (Table 4). In all correlations tested, BF10 values > 100 indicated that there was strong evidence to support an alternative hypothesis for H0. In the control group, a negative correlation was observed for fT4 and U-Cd. Again, BF10 values > 100 supported the alternative hypothesis.

**Figure 2:**
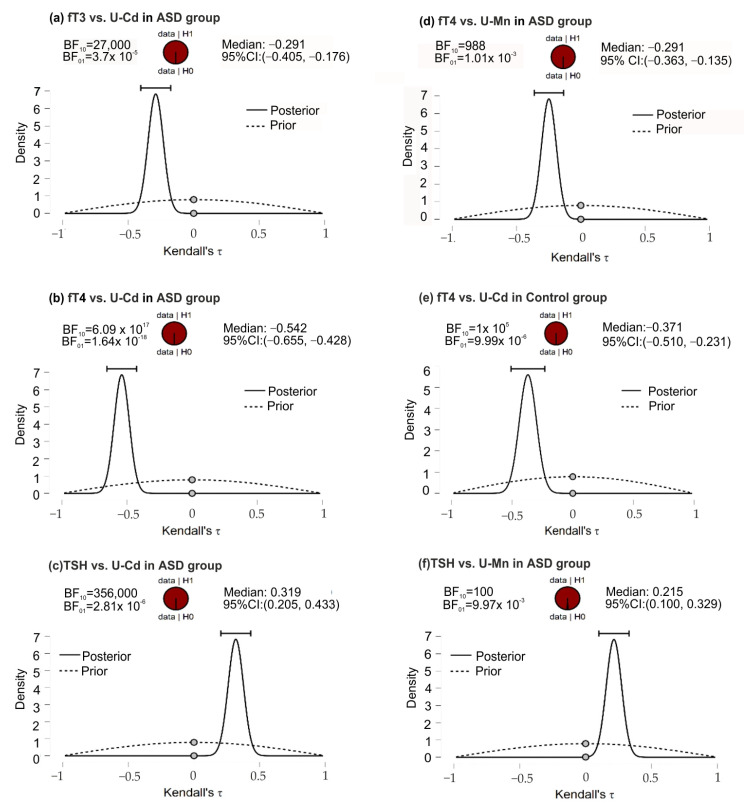
The posterior-prior plots for significant correlations of U-Mn and U-Cd with biochemical variables in the ASD and control groups; (**a**) significant correlations between fT3 and U-Cd in ASD group; (**b**) significant correlations between fT4 and U-Cd in ASD group; (**c**) significant correlations between TSH and U-Mn in ASD group; (**d**) significant correlations between fT4 and U-Mn in ASD group; (**e**) significant correlations between fT4 and U-Cd in control group; (**f**) significant correlations between TSH and U-Cd in ASD group. The dashed lines—prior distribution; the solid lines—posterior distribution; the grey dots—the density values of the prior and posterior distributions; the horizontal bar—95% credible intervals around the median correlation; BF10 and BF01—the Savage−Dickey density ratio depending which way around division is done. Abbreviations: ASD, autism spectrum disorders; fT3, free triiodothyronine; fT4, free thyroxine; TSH, thyroid-stimulating hormone; U-Mn, urinary manganese; U-Cd, urinary cadmium, BF, Bayes Factor.

In the ASD group, the posterior-prior plots, assuming negative correlations of fT3 and U-Cd, fT4 and U-Mn, and fT4 and U-Cd, presented the data fully distributed to the left of 0, with correlation medians −0.291, −0.249, and −0.542, respectively (Figure 2). The higher position of the gray dots (at the 0.0 effect size) on the prior distribution than the posterior distribution indicated BF_10_ support for the alternative hypothesis. In this group of patients, opposite although equally strong support for H1 was noted for the correlation of TSH and U-Mn, TSH, and U-Cd. In the control group, for the fT4 and U-Cd correlations, the full distribution of data was observed to the left of 0, with a median correlation of −0.371 and a Bayes Factor value more strongly supporting H1 than H0.

**Figure 3:**
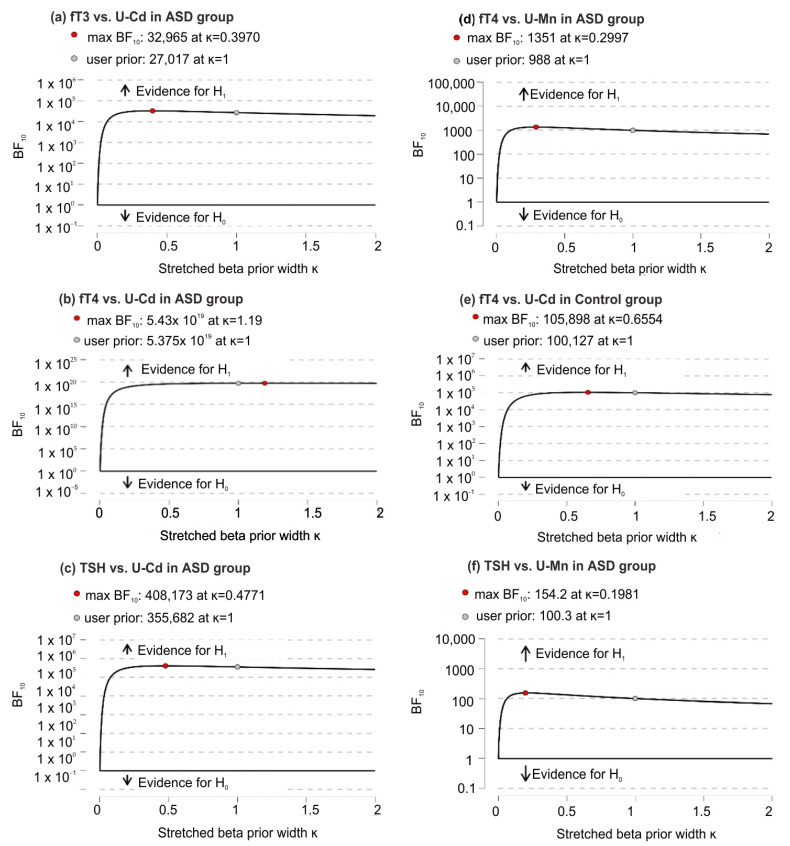
Bayes Factor robustness analysis for significant correlations of U-Mn and U-Cd with biochemical variables in the ASD and control groups; (**a**) significant correlations between fT3 and U-Cd in ASD group; (**b**) significant correlations between fT4 and U-Cd in ASD group; (**c**) significant correlations between TSH and U-Cd in ASD group; (**d**) significant correlations between fT4 and U-Mn in ASD group; (**e**) significant correlations between fT4 and U-Cd in control group; (**f**) significant correlations between TSH and U-Mn in ASD group. Abbreviation: fT3, free triiodothyronine; fT4, free thyroxine; TSH, thyroid-stimulating hormone; U-Mn, urinary manganese; U-Cd, urinary cadmium; BF10, Bayes Factor in favor of H1 over H0 hypothesis.

Based on robustness analysis, it was found (except for small prior widths) that there were very small changes in BF_10_ that consistently supported strong evidence in favor of H1 over H0 (Figure 3).

Detailed analysis of the obtained results based on the age dependencies of the studied patients showed that among individuals aged 14 years and above, there is a statistically significant change between U-Cd in relation to fT4 and fT3. This means that the amount of Cd ions in urine decreased with increasing serum fT4 and fT3 values. A similar relationship for fT4 was also observed among adolescents, both for Cd and Mn ions. There were also significant differences in TSH levels among group age ≥ 14 in relation to U-Cd and U-Mn (positive correlation) and fT4/fT3 in relation to U-Cd in patients over 14 years of age (Table 3).

The second analyzed parameter concerned the sexual differences between the presence of U-Mn and U-Cd and selected biochemical parameters and also showed many significant relationships. Among males, a significant negative correlation was found between the concentration of Mn ions in the urine and the amount of fT4 in the serum as well as a positive correlation with the level of TSH, which was not observed among the examined females. It was also shown that the amount of Cd in urine in males negatively correlates with fT3 and fT4 and positively with fT3/fT4 and TSH. Among the females, only a negative correlation was found in the case of fT4 and U-Cd. This means that males with ASD are more sensitive to Cd and Mn levels than females (Table 3).

Three positive correlations were found in the control group, namely that between fT3/fT4 and U-Cd in the entire study group, between fT3/fT4 and U-Cd in the age ≥14 group, and between fT3/fT4 and U-Cd in male. However, the observed values of the correlation were lower than those observed in patients with ASD in the case of the entire study group and among males by 22.41% and 5.72%, respectively, while higher by 44.18% in the case of the age ≥ 14 group. There were also three negative correlations, namely that between fT4 and U-Cd in the entire study group, among adolescents over 14 years of age, and among males. However, the obtained correlation values were lower in relation to the group of ASD patients by 30.99%, 26.36%, and 19.57%, respectively.

### 3.4. Analysis of the Correlation between Childhood Autism Rating Scale and the Level of U-Mn and U-Cd and Biochemical Parameters in Patients with ASD and Control Group

**Figure 4:**
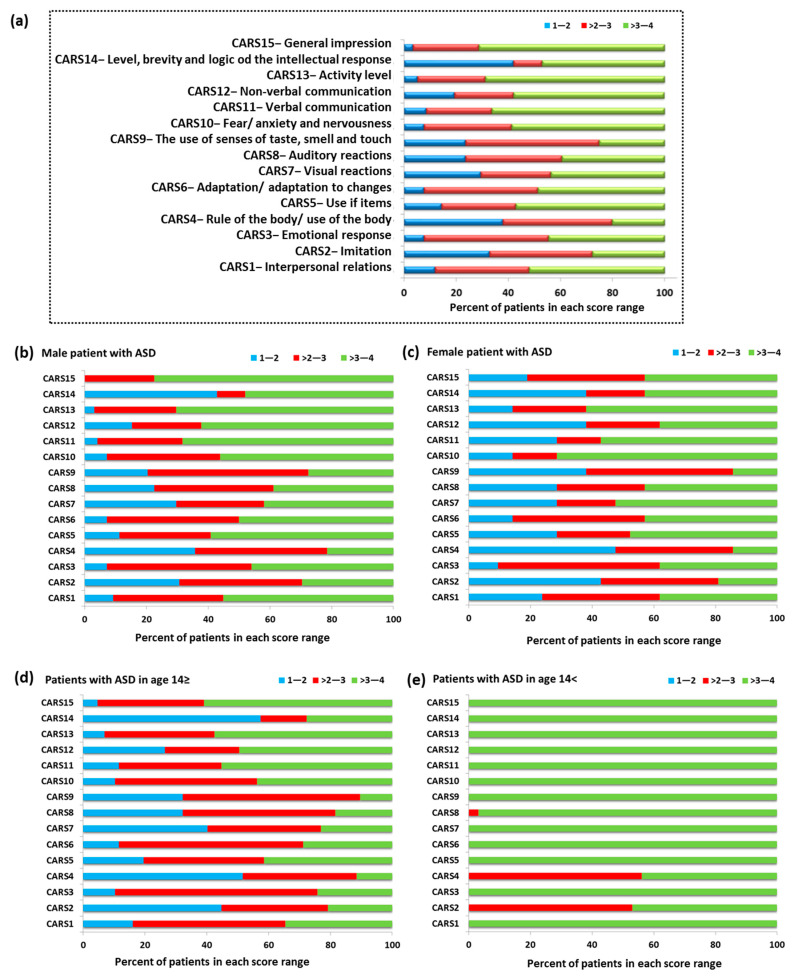
The percentage of patients with ASD in the individual scoring ranges based on CARS; (**a**) percent of patients with ASD in the individual scoring ranges based on CARS in whole tested group; (**b**) percent of patients with ASD in the individual scoring ranges based on CARS in group of male patients; (**c**) percent of patients with ASD in the individual scoring ranges based on CARS in group of female patients; (**d**) percent of patients with ASD in the individual scoring ranges based on CARS in patients with ASD in age ≥ 14; (**e**) percent of patients with ASD in the individual scoring ranges based on CARS in patients with ASD in age < 14.

Another examined aspect of our study concerned the assessment of ASD patients using the Childhood Autism Scale (CARS). CARS consists of 14 areas (CARS 1–14) assessing children’s behavior related to relationships with people, imitating others, adaptation to changes, and verbal and non-verbal communication (Figure 4). The final area of CARS15 is the assessment of the child’s overall impression of behavior. Each of the above-mentioned areas is assessed on a four-point scale, for which 1—the child’s behavior is appropriate for the age (score 1.5), 2—the child’s reactions are slightly abnormal (score 2.5), 3—the child’s reactions are moderately abnormal (score 3.5), and 4—where the child’s reactions are very abnormal (score 4). Obtaining higher scores by a child is associated with a higher level of impairment [55,56]. The percent of patients with ASD in the individual scoring ranges showed that 73.33% of the study group had the highest score in the range > 3–4. This division included 11 CARS categories: CARS1 (52.11%), CARS5 (57.14%), CARS6 (48.74%), CARS7 (43.7%), CARS8 (39.5%), CARS10 (58.83%), CARS11 (66.39%), CARS12 (57.99%), CARS13 (68.91%), CARS14 (47.06%), and CAS15 (71.43%). The remaining 26.67% (four CARS categories) of patients had the highest score in the range > 2–3; this includes the following categories: CARS2 (52.11%), CARS3 (47.90%), CARS4 (42.02%), and CARS9 (51.26%) (Figure 4a).

Detailed analysis of individual age categories and sexes confirmed the tendency observed for the entire research group. In the case of males, 60% of the CARS category had the highest percentage in the range > 3–4, 26.66% in the range > 2–3, and 6.67% in the range 1–2. For the CARS8 category, equal results were also recorded in the ranges > 2–3 and > 3–4 (Figure 4b). In the case of females, 53.34% of the results for each CARS category were in the range > 3–4, 13.33% each for the range 1–2 and > 2–3, and for 20% the results were equal in the range > 2–3 and > 3–4: CARS1, CARS6, and CARS12 (Figure 4c). The differentiation of age categories showed the dominance of the range > 2–3 for most CARS categories in the group of individuals up to 14 years of age (40%), while in the case of adolescents, the range was > 3–4 (86.66%) (Figure 4d,e). Detailed data on the average level of response of ASD patients in each CARS category are presented in Table 1.

Based on the obtained results, an analysis of the correlation between the CARS score and the presence of U-Mn, U-Cd, and the amount of fT3, fT4, and TSH in patients with ASD was performed. Significantly positive correlations of CARS7 (visual reaction) with fT3 and fT4 and negative correlations with TSH were examined. In all cases discussed, BF_10_ values > 100 consistently supported strong evidence in favor of H1 over H0 (Table 5).

Detailed analysis based on age categories showed that among adolescents over 14 years of age, there are positive correlations between CARS7 and fT3 and fT4 levels and a negative correlation with U-Cd and serum TSH levels. Additionally, a statistically significant correlation was found between CARS10 (fear) and the level of TSH (Table 6). The correlations demonstrated in this subgroup were not confirmed for younger patients from the group below 14 years of age. Here, only a positive correlation was observed between CARS9 (use of sense) and the level of TSH (Table 6).

In the case of analyses of the collected data in the context of sex differences, it was shown that in males, there is a statistically significant negative correlation between CARS2 (imitation) and the fT3/fT4 ratio and changes in CARS7, which correlate positively with fT3 and fT4 and negatively with TSH, which we did not observe among the female group. In the group of examined females, only a moderately positive correlation was found between CARS10 and the level of fT3 (Table 7). There were no statistically significant changes between CARS categories and the level of U-Mn in all analyzed groups.

## 4. Discussion

Heavy metals in the environment can have a significant impact on the health and development of the human body. Prenatal exposure to toxic metals or changes in maternal essential elements during pregnancy may be a risk factor for neurodevelopmental disorders, such as attention deficit hyperactivity disorder (ADHD) and ASD, in offspring [57,58,59,60,61,62]. In studies by Skogheim et al. [63], it was checked whether maternal levels of toxic metals and essential elements measured at mid-pregnancy, both individually and as mixtures, were associated with the diagnosis of ADHD or ASD in childhood. The results obtained by the researchers indicate that there is a positive correlation between the levels of metals and toxic elements during pregnancy and the prevalence of ASD and ADHD in children. The most important were arsenic, cadmium, copper, mercury, manganese, magnesium, and lead. Researchers suggest that the levels of these elements may have a negative effect on the development of the nervous system [58,59]. However, throughout his or her life, a person is exposed to contamination and accumulation of heavy metals in the body due to anthropogenic human activity and the presence of these elements in almost every sphere of life. It has been suggested that children with autistic spectrum may have poor heavy-metal-detoxifying mechanisms [59].

Research in recent years has shown that dysregulation of micronutrient homeostasis and increased body contamination with heavy metals, such as lead or mercury, can significantly affect the development of ASD. Therefore, scientists are increasingly eager to focus on researching new elements that may be involved in the development of these diseases, and manganese and cadmium are of particular importance. These metals show higher toxicity in children than in adults, which affects the developing body and may predispose to increased incidence of certain diseases. Due to the ubiquitous presence of manganese in human life and its pro-health aspects, which we mentioned earlier, in the literature, one can find numerous studies measuring its level in various body fluids, such as blood (in the range of 4–15 µg/L), red blood cells, serum (in the range 0.4–0.85 µg/L), urine (in the range 1–8 µg/L), tooth enamel, and hair, in both healthy and ASD children [60].

In literature data regarding excretion of the studied elements, both Cd and Mn may be considered as contradictory. Several studies reported higher urinary excretion levels of Cd in healthy children than in ASD group [58,59]. On the other hand, according to Akyuzlu et al., of findings U-Cd were higher in individuals with ASD [61], which is consistent with our results. The opposite results were achieved for U-Mn, which was lower in cited studies and higher in those with ASD in comparison with the healthy group. This is may be due not only to methodological differences or different research material but also to etiological or geographical differences. Each region of the world will be characterized by completely different contamination of soil, air, or water with these elements, which in turn will also translate into a variable diet in these people (cultural diversity and availability of certain foods and their amount and degree of contamination). In addition, the excretion of some metals varies with age and gender (see Table 1).

At the core of Mn-induced neurotoxicity is the inactivation of the mitochondria, resulting in an increase in reactive oxygen species in brain regions affected by contamination and oxidative stress induction. The exact molecular mechanisms influencing the homeostasis of Mn ions in the human body still remain in the area of intense research. Currently, several transporters responsible for this process are known, including ferroportin, ZIP8/14, glutamate receptor, manganese citrate transporter, DMT1, and CA2 + channels as well as SCL3A10 and SCL39A14, which have a direct impact on the control of manganese levels in the blood, manifesting from not only the presence of these transporters but also the effect on maintaining homeostasis. One should also take into account genetic differences, including the phenomenon of polymorphism, which will condition the proper functioning of these structures [19,64].

In addition, many scientific studies argue that the pathomechanism of diseases caused by manganese contamination in humans is closely correlated with the functioning of the thyroid gland. SCL3A10 and SCL39A14 transporters are responsible for the uptake and release of manganese ions in the body, the mutations of which result in the loss of their functions, which in turn translates into hereditary toxicity of manganese. It manifests itself not only in increased levels of manganese in the blood and brain but also in the thyroid gland and liver, which contributes to the development of neurotoxicity. Studies by Liu and colleagues have shown that the transport activities of these proteins are essential for the proper excretion of manganese by the liver [64]. Changes in SLC3A10 resulted in increased amounts of manganese in the thyroid compared to the SLC39A14 knock-out and knock-out of both transporters, which showed lower levels of manganese and normal thyroid activity. In addition, it was shown that intrathyroid levels of thyroxine with a single SLC30A10 knockout were higher than in the control group, meaning that the hypothyroid phenotype induced by these changes is induced by high levels of manganese in the thyroid, which blocks the production of thyroxine. In our studies, we showed that there is a negative correlation between U-Mn and serum fT4 levels among ASD patients, which was not found in the control group [19]. Accumulation of manganese in patients with specific ASD features as measured by the Autism Diagnostic Observation Schedule (ADOS) was the subject of a study by Fiore et al. [65]. They showed that the level of manganese in the hair was inversely correlated with the cognitive level (full IQ) of people with ASD. These studies confirm the significant influence of manganese in neuronal development. Additionally, another study related to the analysis of primary incisors in children six to nine years of age showed significant differences in the concentration of metals in the tooth samples of children with ASD and typically developing children. The content of manganese was three times higher in the teeth of children with ASD compared to children from the control group [66]. In the light of the above varied data obtained in studies by various teams and taking into account the potential environmental differences related to exposure to heavy metals, it is difficult to indicate the unequivocal effect of manganese on human health. Moreover, reference values for Mn of the 24-h urine measurement have not been established for patients <18 years of age.

As regards the second analyzed element, which was cadmium (Cd), we also observed significant correlations that may affect the development of ASD in adolescents. Cadmium alone has multiple effects on cells; therefore, it can directly or indirectly interfere with the development of the brain. It disrupts the progress of the cell cycle, proliferation, differentiation, and the induction of apoptosis. The excess of this heavy metal (as much as 50% of cadmium absorbed by the body) accumulates in the liver and kidneys, where its half-life in the body is 6–38 years and 4–19 years, respectively [17]. It has also been shown that this element can accumulate in bones and the entire skeletal system as well as in the brain, as is the case with zebrafish [67]. The problem of the influence of cadmium on the human body is still a subject of intense research by many scientists. Similar to the data on manganese concentrations in the human body, the concentration of Cd among ASD patients reported in the literature is also extremely varied, and the reference values for Cd of the 24-h urine measurement have not been established for patients <18 years of age as well. The largest amount of data concerns the presence of this element in the hair, which, depending on the researcher, is 0.021 µg/g [68], 0.0277 µg/g [43], or even 0.62 mg/kg [58].

In the case of autism-related research, the hypothesis we adopted was the effect of metals on thyroid function. Indications that Cd may affect thyroid function were found by the team of Chen et al. [68], who analyzed National Health and Nutrition Examination Survey (NHANES) data for 2007–2008 obtained from the general U.S. population with higher levels of environmental exposure. Analyses showed that higher levels of thyroid hormones and thyroglobulin (Tg) in adults correlated with increased levels of urinary Cd, i.e., greater exposure to Cd. However, in terms of TSH level itself, it was no longer associated with exposure to Cd. Consequently, this raises questions about the effects of this metal on the thyroid gland [69]. Another analysis of NHANES data from 2007–2008 confirmed that urinary Cd levels also correlated with increased T3 and T4 levels. In addition, studies have shown elevated blood Cd levels were associated with decreased TSH levels, suggesting that this inverse relationship between Cd and TSH is indicative of overt thyroid disease caused by exposure to Cd. Another aspect of the observation was that the presence of blood Cd correlated with lower TSH levels and unchanged levels of T3 and T4, indicating the presence of subclinical primary hyperthyroidism in this case. On the other hand, urinary Cd levels are associated with higher T3 and T4 levels, but TSH levels remained unchanged, indicating secondary hyperthyroidism. Additionally, the data obtained from NHANES were used to analyze the correlation between the presence of Cd in urine and the concentration of thyroid hormones [68]. The presence of Cd is different both in human blood and urine and may indicate different levels of contamination in the whole organism. Research suggests that urinary Cd excretion is a function of total body content of Cd, nephron numbers, tubular reabsorption capacity, and the presence of other disease, and other conditions, such as hypertension [70]. These findings led to the hypothesis that thyroid hormone levels are strongly related to the total body burden on Cd, while the observed changes in TSH levels alone may reflect the relatively recent exposure to this metal. Currently, researchers are trying to consider the effects of interactions between various trace and toxic metals. One publication found positive statistically significant correlations between Cd and fT3 levels and Cd and Tg levels but only for men, indicating gender differences in thyroid function response to Cd exposure. The evidence for a link between Cd and thyroid dysfunction remains inconclusive, and more research is needed to clarify the problem and better understand the effects of Cd on the thyroid gland [71,72,73].

Some studies cited in the introduction suggest that lower production of thyroid hormones at birth may influence neurodevelopmental impairment (subsequently diagnosis of ASD) that persists even though the hormone levels return later to normal ranges (it is worth noting that this is a fairly broad range: TSH from 0.45–5.0 mIU/L under 14 y and from 0.4 to 4.0 mIU/L above 14 y [52]) [31]. Frye at al. emphasized the impact of folate receptor autoantibodies, which may contribute to the higher TSH levels in children with autism [32].

However, all the studies conducted, including ours, did not test the interaction of the environment with the genetic predispositions and conditions of the studied children and adolescents. Therefore, as researchers around the world argue, analyzing and comparing the presented data without taking into account the gene–environment interaction is incomplete and cannot be interpreted as unrelated.

Results of correlation analysis of U-Mn, U-Cd, and biochemical variables with CARS scores in group of patients based on their sex (Table 7) revealed a link with visual responsiveness. There is growing evidence for disruption of the auditory and visual processing pathways in autism [74]. Individuals with autism tend to manifest significant disturbances in perception and processing of sensual stimuli from the environment as well as from their own body. Both over- and under-sensitivity is characteristic for the described neurodevelopmental disorder. A marked arousal when exposed to strong visual stimuli is usually observed, and response depends on the type of visual information [74]. Both autism and visual problems can cause an avoidance of eye contact or problems with visual attention, etc. Hypersensitivity to light and other visual stimuli is a very common problem in ASD.

An example of animal-based research showed interesting effects of cadmium pollution on the vision of adult zebrafish Danio rerio [75]. The results demonstrated that cadmium impairs functional responses, particularly through an increase in light sensitivity. At this point, however, it is difficult to prove that some kind of a photosensitivity in autistic individuals confirmed by the diagnostic tests is related to cadmium exposure and its effects on retina. In the light of some human studies that determined Cd accumulation in human retinal tissues, specifically the neural retina, retinal pigment epithelium, and choroid [76], it seems to be justified to conduct studies on cadmium effects in the population with autism. Recently, studies have been undertaken on the effects of other toxic metals on the retina of donors without visual impairment and no significant retinal abnormalities [77]. It was confirmed that toxic metals accumulate in the retina and may contribute to the pathogenesis of visual disturbances.

If an association between increased cadmium levels in individuals with ASD and its effects on increased photosensitivity could be proven, preventive measures could be taken early in the patients’ lives. Future larger studies should focus on confirming whether Cd is associated with disturbed visual responsiveness in ASD and on the explanation of why this relationship is evident in the older group and not in the younger group (Table 6). Undoubtedly, our study confirms that elemental homeostasis and toxin exposure affect the severity of specific autistic symptoms.

A strong positive correlation between BMI and cadmium concentration, TSH, and once fT3/fT4 was found in our studies. These correlations show that the higher the BMI value, the higher the concentration of U-Cd and the concentration of thyroid hormones (Table 2). The literature data show a relationship between U-Cd levels and obesity. Studies by Green et al. [78] indicate that there is a relationship between the presence of cadmium in the blood of pregnant women and an increased risk of adolescent obesity in offspring obesity. Additionally, this trend was tested in an animal model (zebrafish), in which exposure to Cd was also associated with increased obesity [79]. On the other hand, in the case of the influence of BMI on the thyroid gland, studies were also carried out on women, in which high maternal BMI coincides with the immature histological phenotype, affecting the thyroid gland of female fetuses [80]. In turn, another study related to the Korean population showed that the risk of an underactive thyroid gland increased gradually with blood cadmium content in men, while no such correlation was observed in the case of women [76].

Despite the above information, we have not encountered any studies that directly refer to the correlation between the concentration of U-Cd, thyroid hormones, and BMI.

### Advantages and Limitations of the Study

A detailed analysis of individual subgroups of patients depending on age and sex allowed for a better and more complete picture of the studied patients and the relationships between the concentration of U-Cd and U-Mn and selected biochemical parameters of thyroid function.

The limitations of the present study refer to the missing information on the intake of micronutrients and, generally, on dietary factors that could affect relationships between both toxic and essential elements. As it is well known, chronic exposure to the toxic metals is particularly dangerous to the body. Since urine testing can only show recent exposure, consideration of other types of samples, such as blood, hair, or fingernails, would add to the knowledge on the discussed subject. However, in our study, the non-invasiveness of sampling was crucial for project participants. It is worth mentioning that hormone tests are usually done prophylactically, and metal analysis is not a routinely ordered test.

Although thyroid hormone levels were examined, and no serious pathology of this gland was found in either group of our study participants, additional imaging studies (e.g., ultrasonography) could bring further information about the location, size, and echostructure of the thyroid gland as well as its vascularity at the time of testing. Another limitation of this study is the inability to relate genetic interactions with the patients’ living environment.

## 5. Conclusions

The conducted studies showed a significant correlation between the studied biochemical parameters of thyroid gland and the BMI index and the concentration of Mn and Cd in the urine. Among the entire group of ASD patients, we found a positive correlation between BMI and U-Cd and TSH and fT3/fT4 as well as a negative correlation between the concentration of fT3 and fT4 in the serum. In addition, we showed a number of changes in the studied parameters in relation to sex and age differences of the studied patients. We also found a negative correlation for fT3 and U-Cd, fT4 and U-Mn, and fT4 and U-Cd and a positive correlation for TSH and U-Mn as well as TSH and U-Cd in the entire group of patients with ASD as well as changes in gender and age of the patients.

Correlation analysis between CARS results and the content of both Mn in urine and Cd in urine as well as fT3, fT4, and TSH values in ASD patients showed significantly positive correlation of CARS7 (visual reaction) with fT3 and fT4 and a negative correlation with TSH for the whole study group. In addition to the CARS7-related relationships, detailed analysis showed a negative correlation between CARS2 and the fT3/fT4 ratio among males with ASD and a positive correlation between CARS10 and fT3 levels among females. In the group of adolescents over 14 years of age, it was also observed that CARS10 negatively correlates with serum TSH levels, and among younger individuals, CARS9 positively correlates with TSH.

Additional studies are required to identify the long-term effects of the interaction of Mn and Cd in ASD and to reveal the mechanisms underlying the observed associations between symptom severity and Mn and Cd excretion.

## Figures and Tables

**Table 1:** Detailed data on the mean, standard deviation, median, and range level of response of ASD patients in individual CARS *.

Scores	1–2		>2–3		>3–4		For Total Group (*n* = 119)	
	Mean ± SD	Median (Range)	Mean ± SD	Median (Range)	Mean ± SD	Median (Range)	Mean ± SD	Median (Range)
CARS1	2.00 ± 0.00	2.00 (2.0–2.0)	2.78 ± 0.25	3.00 (2.5–3.0)	3.75 ± 0.25	3.50 (3.5–4.0)	3.18 ± 0.66	3.5 (2.0–4.0)
CARS2	2.00 ± 0.00	2.00 (2.0–2.0)	2.86 ± 0.23	3.00 (2.5–3.0)	3.59 ± 0.20	3.50 (3.5–4.0)	2.78 ± 0.65	3.0 (2.0–4.0)
CARS3	1.89 ± 0.22	2.00 (1.5–2.0)	2.93 ± 0.18	3.00 (2.5–3.0)	3.81 ± 0.24	4.00 (3.5–4.0)	3.24 ± 0.61	3.0 (1.5–4.0)
CARS4	1.76 ± 0.25	2.00 (1.5–2.0)	2.81 ± 0.25	3.00 (2.5–3.0)	3.69 ± 0.25	3.50 (3.5–4.0)	2.59 ± 0.77	2.5 (1.5–4.0)
CARS5	1.94 ± 0.17	2.00 (1.5–2.0)	2.81 ± 0.25	3.00 (2.5–3.0)	3.90 ± 0.20	4.00 (3.5–4.0)	3.31 ± 0.77	3.5 (1.5–4.0)
CARS6	1.56 ± 0.17	1.50 (1.5–2.0)	2.99 ± 0.07	3.00 (2.5–3.0)	3.60 ± 0.20	3.50 (3.5–4.0)	3.18 ± 0.57	3.0 (1.5–4.0)
CARS7	1.54 ± 0.14	1.50 (1.5–2.0)	2.75 ± 0.25	2.75 (2.5–3.0)	3.68 ± 0.24	3.50 (3.5–4.0)	2.80 ± 0.93	3.0 (1.5–4.0)
CARS8	1.96 ± 0.13	2.00 (1.5–2.0)	2.89 ± 0.21	3.00 (2.5–3.0)	3.87 ± 0.22	4.00 (3.5–4.0)	3.06 ± 0.77	3.0 (1.5–4.0)
CARS9	1.95 ± 0.16	2.00 (1.5–2.0)	3.00 ± 0.00	3.00 (3.0–3.0)	3.83 ± 0.24	4.00 (3.5–4.0)	2.96 ± 0.68	3.0 (1.5–4.0)
CARS10	1.78 ± 0.26	2.00 (1.5–2.0)	2.99 ± 0.08	3.00 (2.5–3.0)	3.89 ± 0.21	4.00 (3.5–4.0)	3.42 ± 0.66	3.5 (1.5–4.0)
CARS11	1.85 ± 0.24	2.00 (1.5–2.0)	2.95 ± 0.15	3.00 (2.5–3.0)	3.90 ± 0.20	4.00 (3.5–4.0)	3.49 ± 0.67	4.0 (1.5–4.0)
CARS12	1.91 ± 0.19	2.00 (1.5–2.0)	2.87 ± 0.22	3.00 (2.5–3.0)	3.75 ± 0.25	4.00 (3.5–4.0)	3.20 ± 0.76	3.5 (1.5–4.0)
CARS13	1.89 ± 0.26	2.00 (1.5–2.0)	2.90 ± 0.20	3.00 (2.5–3.0)	3.82 ± 0.24	4.00 (3.5–4.0)	3.48 ± 0.60	3.5 (1.5–4.0)
CARS14	1.92 ± 0.23	2.00 (1.0–2.0)	2.96 ± 0.14	3.00 (2.5–3.0)	3.65 ± 0.23	3.50 (3.5–4.0)	2.85 ± 0.85	3.0 (1.0–4.0)
CARS15	1.75 ± 0.29	1.75 (1.5–2.0)	2.87 ± 0.22	3.00 (2.5–3.0)	3.81 ± 0.25	4.00 (3.5–4.0)	3.50 ± 0.57	3.5 (1.5–4.0)
Total CARS							47.05 ± 5.57	48.0 (30.0–59.0)

* Due to missing data (*n* = 10) in CARS variables, the analyses were based on data from 119 autistic participants.

**Table 2:** Basic characteristics of ASD and the control group.

Group	Descripted Data	ASD Patients		Control		*t* Statistic	*p*-Value
		Mean ± SD	Median (Min–Max)	Mean ± SD	Median (Min–Max)		
Total	Age (years)	14.1 ± 1.4	14.3 (12.1–17.0)	14.7 ± 1.2	14.6 (12.1–16.8)	−2.9677	0.0033 *
	BMI (kg/m^2^)	23.7 ± 3.2	24.2 (16.8–31.1)	21.0 ± 1.4	20.8 (18.7–24.8)	7.4215	<0.0001 *
	fT3 (pmol/L)	5.4 ± 1.0	5.2 (3.8–8.1)	5.4 ± 0.7	5.2 (4.2–6.7)	−0.2426	0.8086
	fT4 (pmol/L)	17.2 ± 4.2	16.6 (9.9–26.5)	16.8 ± 3.0	16.5 (11.3–24.3)	0.6805	0.4969
	fT3/fT4	0.32 ± 0.07	0.32 (0.27–0.37)	0.33 ± 0.07	0.32 (0.28–0.37)	−0.4522	0.6516
	TSH (mIU/L)	2.4 ± 1.4	2.1 (0.5–5.2)	1.7 ± 1.0	1.5 (0.2–4.3)	3.6198	0.0004 *
	U-Mn (µg/d)	0.043 ± 0.026	0.036 (0.007–0.133)	0.012 ± 0.002	0.012 (0.008–0.022)	10.9764	<0.0001 *
	U-Cd (µg/d)	0.499 ± 0.359	0.546 (0.011–1.326)	0.127 ± 0.163	0.071 (0.003–0.680)	8.9985	<0.0001 *
Age ≥ 14	Age (years)	15.2 ± 0.8	14.9 (14.1–17.0)	15.3 ± 0.8	15.2 (14.1–16.8)	−0.732	0.4655
	BMI (kg/m^2^)	24.0 ± 3.0	24.2 (17.3–31.1)	20.9 ± 1.3	20.8 (18.7–23.5)	7.479	<0.0001 *
	fT3 (pmol/L)	5.4 ± 1.1	5.2 (3.8–8.1)	5.4 ± 0.7	5.3 (4.2–6.7)	0.168	0.8667
	fT4 (pmol/L)	16.7 ± 4.2	16.5 (9.9–26.5)	16.9 ± 3.3	16.6 (11.3–24.3)	−0.297	0.7672
	fT3/fT4	0.3 ± 0.1	0.3 (0.2–0.6)	0.3 ± 0.1	0.3 (0.2–0.6)	0.462	0.6448
	TSH (mIU/L)	2.3 ± 1.4	2.1 (0.5–5.0)	1.4 ± 0.8	1.2 (0.2–3.5)	4.369	<0.0001 *
	U-Mn (µg/d)	0.04 ± 0.03	0.04 (0.01–0.13)	0.01 ± 0.0	0.01 (0.01–0.02)	9.031	<0.0001 *
	U-Cd (µg/d)	0.53 ± 0.36	0.59 (0.01–1.19)	0.14 ± 0.17	0.08 (0–0.68)	7.522	<0.0001 *
Age < 14	Age (years)	12.6 ± 0.5	12.4 (12.1–13.8)	13.4 ± 0.7	13.7 (12.1–13.9)	−5.873	<0.0001 *
	BMI (kg/m^2^)	23.2 ± 3.5	24.3 (16.8–29.8)	21.1 ± 1.7	20.8 (18.7–24.8)	3.081	0.0028
	fT3 (pmol/L)	5.3 ± 0.9	5.1 (4.0–8.1)	5.4 ± 0.7	5.2 (4.3–6.6)	−0.550	0.5838
	fT4 (pmol/L)	17.8 ± 4.3	17.2 (9.9–25.6)	16.6 ± 2.3	16.3 (12.6–20.5)	1.383	0.1704
	fT3/fT4	0.3 ± 0.1	0.3 (0.2–0.6)	0.3 ± 0.1	0.3 (0.2–0.5)	−1.135	0.2598
	TSH (mIU/L)	2.5 ± 1.5	2.1 (0.5–5.2)	2.4 ± 1.0	2.6 (0.4–4.3)	0.197	0.8442
	U-Mn (µg/d)	0.04 ± 0.03	0.03 (0.01–0.1)	0.01 ± 0.0	0.01 (0.01–0.02)	6.243	<0.0001 *
	U-Cd (µg/d)	0.46 ± 0.35	0.45 (0.02–1.33)	0.1 ± 0.14	0.06 (0.01–0.57)	5.134	<0.0001 *
Male	Age (years)	14.0 ± 1.4	14.3 (12.1–17.0)	14.7 ± 1.2	14.7 (12.1–16.7)	−2.851	0.0049 *
	BMI (kg/m^2^)	23.9 ± 3.2	24.5 (16.8–30.8)	21.2 ± 1.4	21.3 (18.7–24.1)	5.751	<0.0001 *
	fT3 (pmol/L)	5.3 ± 1.0	5.1 (3.8–8.1)	5.2 ± 0.6	5.2 (4.4–6.7)	0.381	0.7036
	fT4 (pmol/L)	16.9 ± 4.3	16.5 (9.9–26.5)	16.6 ± 2.7	16.4 (11.3–24.1)	0.437	0.6626
	fT3/fT4	0.3 ± 0.1	0.3 (0.2–0.6)	0.3 ± 0.1	0.3 (0.2–0.4)	0.389	0.6980
	TSH (mIU/L)	2.5 ± 1.5	2.2 (0.5–5.2)	1.7 ± 1.0	1.5 (0.2–4.3)	3.539	0.0005 *
	U-Mn (µg/d)	0.04 ± 0.03	0.03 (0.01–0.13)	0.01 ± 0.01	0.01 (0.01–0.02)	8.379	<0.0001 *
	U-Cd (µg/d)	0.53 ± 0.36	0.57 (0.01–1.33)	0.15 ± 0.19	0.07 (0.01–0.68)	7.305	<0.0001 *
Female	Age (years)	14.4 ± 1.5	14.4 (12.1–16.7)	14.6 ± 1.2	14.2 (12.1–16.8)	−0.574	0.5687
	BMI (kg/m^2^)	23.0 ± 3.3	22.8 (17.8–31.1)	20.5 ± 1.3	20.4 (18.7–24.8)	3.743	0.0005 *
	fT3 (pmol/L)	5.7 ± 1.0	5.3 (4.2–8.1)	5.6 ± 0.8	5.7 (4.2–6.7)	0.092	0.9270
	fT4 (pmol/L)	18.3 ± 4	19.5 (12.6–25.6)	17.1 ± 3.4	16.5 (11.3–24.3)	1.271	0.2094
	fT3/fT4	0.3 ± 0.1	0.3 (0.2–0.6)	0.3 ± 0.1	0.3 (0.2–0.6)	−0.950	0.3466
	TSH (mIU/L)	2.0 ± 1.2	1.9 (0.7–4.5)	1.9 ± 1.0	1.8 (0.3–3.9)	0.536	0.5944
	U-Mn (µg/d)	0.05 ± 0.02	0.05 (0.02–0.09)	0.01 ± 0.01	0.01 (0.01–0.02)	8.745	<0.0001 *
	U-Cd (µg/d)	0.33 ± 0.31	0.25 (0.01–0.96)	0.09 ± 0.09	0.06 (0.01–0.5)	4.170	0.0001 *

Age ≥ 14: ASD patients = 75, Control = 58; Age < 14: ASD patients = 54, Control = 28; Abbreviations: ASD, autism spectrum disorders; BMI, body mass index; fT3, free triiodothyronine; fT4, free thyroxine; TSH, thyroid-stimulating hormone; U-Mn, urinary manganese (microgram per 24 h); U-CD, urinary cadmium (microgram per 24 h); * statistical significance; *p*-value < 0.05.

**Table 3:** Correlation of BMI with biochemical variables by studied groups.

Group	Variables	Statistics	ASD Patients	Control
			BMI	BMI
Total	U-Mn	Kendall’s tau	0.140	−0.109
		BF_10_	1.821	0.421
	U-Cd	Kendall’s tau	0.294 ***	−0.026
		BF_10_	20,910.305	0.15
	fT3	Kendall’s tau	−0.252 ***	−0.151
		BF_10_	850.492	1.125
	fT4	Kendall’s tau	−0.392 ***	−0.013
		BF_10_	2.566 × 10^8^	0.143
	fT3/fT4	Kendall’s tau	0.225 ***	−0.065
		BF_10_	137.835	0.206
	TSH	Kendall’s tau	0.553 ***	0.17
		BF_10_	4.291 × 10^17^	1.996
Age ≥ 14	U-Mn	Kendall’s tau	0.205	−0.109
		BF_10_	4.203	0.347
	U-Cd	Kendall’s tau	0.301 ***	0.014
		BF_10_	197.826	0.173
	fT3	Kendall’s tau	−0.274 **	−0.15
		BF_10_	57.644	0.657
	fT4	Kendall’s tau	−0.409 ***	−0.038
		BF_10_	86,022.994	0.186
	fT3/fT4	Kendall’s tau	0.192	−0.056
		BF_10_	2.806	0.207
	TSH	Kendall’s tau	0.573 ***	0.109
		BF_10_	3.058 × 10^10^	0.349
Age < 14	U-Mn	Kendall’s tau	0.039	−0.118
		BF_10_	0.192	0.353
	U-Cd	Kendall’s tau	0.268	−0.083
		BF_10_	9.829	0.293
	fT3	Kendall’s tau	−0.238	−0.123
		BF_10_	4.224	0.365
	fT4	Kendall’s tau	−0.356 ***	0.024
		BF_10_	210.095	0.247
	fT3/fT4	Kendall’s tau	0.218	−0.069
		BF_10_	2.523	0.277
	TSH	Kendall’s tau	0.518 ***	0.335
		BF_10_	557,069.220	4.905
Male	U-Mn	Kendall’s tau	0.159	0.066
		BF_10_	2.409	0.225
	U-Cd	Kendall’s tau	0.295 ***	0.011
		BF_10_	3192.671	0.178
	fT3	Kendall’s tau	−0.249 ***	−0.149
		BF_10_	172.382	0.607
	fT4	Kendall’s tau	−0.381 ***	0.017
		BF_10_	2.681 × 10^6^	0.18
	fT3/fT4	Kendall’s tau	0.215 *	−0.103
		BF_10_	27.213	0.32
	TSH	Kendall’s tau	0.554 ***	0.136
		BF_10_	4.067× 10^14^	0.498
Female	U-Mn	Kendall’s tau	0.115	−0.233
		BF_10_	0.360	1.238
	U-Cd	Kendall’s tau	0.134	−0.02
		BF_10_	0.393	0.231
	fT3	Kendall’s tau	−0.270	−0.025
		BF_10_	1.121	0.233
	fT4	Kendall’s tau	−0.408	−0.089
		BF_10_	6.564	0.291
	fT3/fT4	Kendall’s tau	0.201	0.065
		BF_10_	0.602	0.26
	TSH	Kendall’s tau	0.532 **	0.363 *
		BF_10_	59.554	13.741

Abbreviation: ASD, autism spectrum disorders; fT3, free triiodothyronine; fT4, free thyroxine; TSH, thyroid-stimulating hormone; U-Mn, urinary manganese; U-Cd, urinary cadmium; BF_10_, Bayes Factor in favor of H1 over H0 hypothesis; * BF_10_ > 10, ** BF_10_ > 30, *** BF_10_ > 100.

**Table 4:** Correlation of U-Mn and U-Cd with biochemical variables by studied groups.

Group	Variables	Statistic	ASD Patients		Control	
			U-Mn	U-Cd	U-Mn	U-Cd
Total	fT3	Kendall’s tau	−0.147	−0.297 ***	0.044	−0.111
		BF_10_	2.365	27,017.238	0.168	0.436
	fT4	Kendall’s tau	−0.254 ***	−0.555 ***	0.008	−0.383 ***
		BF_10_	988.009	6.089 × 10^17^	0.141	100,126.951
	fT3/fT4	Kendall’s tau	0.124	0.348 ***	−0.015	0.270 ***
		BF_10_	0.982	2.699 × 10^6^	0.144	110.496
	TSH	Kendall’s tau	0.220 ***	0.327 ***	−0.173	−0.065
		BF_10_	100.325	355,682.370	2.216	0.207
Age ≥ 14	fT3	Kendall’s tau	−0.169	−0.441 ***	0.080	−0.064
		BF_10_	1.449	757,743.584	0.250	0.219
	fT4	Kendall’s tau	−0.207	−0.569 ***	−0.039	−0.419 ***
		BF_10_	4.509	1.999 × 10^10^	0.187	6533.091
	fT3/fT4	Kendall’s tau	0.065	0.249 *	0.048	0.359 ***
		BF_10_	0.210	20.403	0.196	398.398
	TSH	Kendall’s tau	0.294 ***	0.385 ***	−0.084	−0.002
		BF_10_	146.050	19,449.984	0.261	0.171
Age < 14	fT3	Kendall’s tau	−0.133	−0.081	−0.035	−0.236
		BF_10_	0.474	0.255	0.251	1.076
	fT4	Kendall’s tau	−0.304 **	−0.537 ***	0.061	−0.348
		BF_10_	30.969	1.702 × 10^6^	0.269	6.183
	fT3/fT4	Kendall’s tau	0.174	0.494 ***	−0.103	0.067
		BF_10_	0.964	137,815.131	0.323	0.274
	TSH	Kendall’s tau	0.114	0.255	−0.156	−0.046
		BF_10_	0.365	6.590	0.468	0.257
Male	fT3	Kendall’s tau	−0.162	−0.288 ***	0.033	−0.164
		BF_10_	2.686	1906.359	0.188	0.791
	fT4	Kendall’s tau	−0.268 ***	−0.562 ***	0.089	−0.452 ***
		BF_10_	536.135	1.178 × 10^15^	0.275	15,527.377
	fT3/fT4	Kendall’s tau	0.126	0.367 ***	−0.079	0.346 ***
		BF_10_	0.791	829,368.688	0.250	138.337
	TSH	Kendall’s tau	0.218 **	0.317 ***	−0.104	−0.057
		BF_10_	32.285	15,238.082	0.324	0.213
Female	fT3	Kendall’s tau	−0.222	−0.133	−0.064	−0.073
		BF_10_	0.715	0.390	0.259	0.269
	fT4	Kendall’s tau	−0.239	−0.563 ***	−0.123	−0.302
		BF_10_	0.828	112.418	0.364	3.938
	fT3/fT4	Kendall’s tau	0.163	0.373	0.004	0.195
		BF_10_	0.464	3.935	0.228	0.749
	TSH	Kendall’s tau	0.337	0.315	−0.387 *	−0.062
		BF_10_	2.409	1.851	24.359	0.258

Abbreviation: ASD, autism spectrum disorders; fT3, free triiodothyronine; fT4, free thyroxine; TSH, thyroid-stimulating hormone; U-Mn, urinary manganese; U-Cd, urinary cadmium; BF_10_, Bayes Factor in favor of H1 over H0 hypothesis; * BF_10_ > 10, ** BF_10_ > 30, *** BF_10_ > 100.

**Table 5:** Correlation between U-Mn, U-Cd, and biochemical variables with CARS scores in whole group of patients.

Variable	Statistic	U-Mn	U-Cd	fT3	fT4	fT3/fT4	TSH
CARS1	Kendall’s tau	−0.078	0.004	−0.040	0.097	−0.118	−0.043
	BF_10_	0.260	0.120	0.147	0.407	0.717	0.153
CARS2	Kendall’s tau	−0.019	−0.070	−0.108	0.081	−0.168	0.029
	BF_10_	0.125	0.227	0.543	0.282	4.449	0.134
CARS3	Kendall’s tau	0.076	0.096	0.006	−0.047	0.043	0.025
	BF_10_	0.252	0.396	0.120	0.159	0.152	0.130
CARS4	Kendall’s tau	−0.079	−0.056	0.031	0.136	−0.128	−0.109
	BF_10_	0.266	0.178	0.136	1.280	0.980	0.546
CARS5	Kendall’s tau	0.040	0.085	−0.059	−0.055	−0.001	0.049
	BF_10_	0.147	0.303	0.188	0.178	0.120	0.164
CARS6	Kendall’s tau	−0.051	−0.035	0.089	0.053	−0.005	−0.079
	BF_10_	0.167	0.140	0.332	0.172	0.120	0.267
CARS7	Kendall’s tau	−0.104	−0.144	0.272 ***	0.271 ***	−0.086	−0.272 ***
	BF_10_	0.481	1.734	1667.124	1548.419	0.313	1654.800
CARS8	Kendall’s tau	−0.104	−0.030	0.066	0.068	−0.005	−0.047
	BF_10_	0.481	0.134	0.209	0.217	0.120	0.160
CARS9	Kendall’s tau	0.104	0.059	−0.078	−0.103	0.061	0.156
	BF_10_	0.479	0.189	0.265	0.474	0.192	2.735
CARS10	Kendall’s tau	−0.026	−0.018	0.053	0.060	−0.006	−0.116
	BF_10_	0.131	0.125	0.172	0.191	0.120	0.680
CARS11	Kendall’s tau	0.017	0.032	0.024	−0.040	0.048	0.009
	BF_10_	0.124	0.137	0.129	0.147	0.160	0.121
CARS12	Kendall’s tau	0.002	0.039	−0.038	−0.044	0.024	0.009
	BF_10_	0.120	0.146	0.144	0.153	0.129	0.121
CARS13	Kendall’s tau	−0.098	0.062	−0.052	−0.015	−0.013	−0.043
	BF_10_	0.411	0.196	0.169	0.123	0.122	0.152
CARS14	Kendall’s tau	−0.038	−0.046	−0.007	−0.007	8.340 × 10^−4^	−0.001
	BF_10_	0.144	0.157	0.121	0.120	0.120	0.120
CARS15	Kendall’s tau	−0.101	0.127	−0.137	−0.054	−0.019	0.077
	BF_10_	0.440	0.941	1.325	0.174	0.125	0.257
Total CARS	Kendall’s tau	−0.060	−0.024	0.017	0.083	−0.060	−0.070
	BF_10_	0.192	0.129	0.124	0.291	0.190	0.226

Due to missing data (*n* = 10) in CARS variables, the analyses were based on data from 119 autistic participants; BF_10_, Bayes Factor in favor of H1 over H0 hypothesis; *** BF_10_ > 100.

**Table 6:** Correlation between U-Mn, U-Cd, and biochemical variables with CARS scores in group of patients based on their age.

Group	Variable	Statistic	U-Mn	U-Cd	fT3	fT4	fT3/fT4	TSH
Age ≥ 14	CARS1	Kendall’s tau	−0.017	−0.050	0.000	0.135	−0.161	−0.103
		BF_10_	0.159	0.187	0.156	0.596	1.041	0.342
	CARS2	Kendall’s tau	0.094	0.019	−0.139	0.024	−0.155	0.049
		BF_10_	0.301	0.160	0.650	0.163	0.913	0.186
	CARS3	Kendall’s tau	0.027	0.118	−0.028	−0.023	0.009	−0.142
		BF_10_	0.164	0.436	0.165	0.162	0.157	0.688
	CARS4	Kendall’s tau	−0.039	−0.184	0.160	0.220	−0.164	−0.163
		BF_10_	0.174	1.869	1.025	5.606	1.135	1.110
	CARS5	Kendall’s tau	0.018	0.069	−0.037	−0.064	0.053	0.056
		BF_10_	0.160	0.222	0.172	0.211	0.191	0.197
	CARS6	Kendall’s tau	−0.075	−0.068	0.164	0.086	0.054	−0.192
		BF_10_	0.235	0.220	1.138	0.268	0.193	2.355
	CARS7	Kendall’s tau	−0.041	−0.255 *	0.329 ***	0.328 ***	−0.115	−0.283 **
		BF_10_	0.176	19.089	444.182	432.963	0.415	57.510
	CARS8	Kendall’s tau	−0.149	−0.076	0.142	0.081	0.064	−0.136
		BF_10_	0.807	0.239	0.685	0.252	0.211	0.612
	CARS9	Kendall’s tau	0.051	0.065	−0.011	−0.032	0.070	0.017
		BF_10_	0.189	0.213	0.157	0.168	0.222	0.159
	CARS10	Kendall’s tau	−0.035	−0.127	0.185	0.144	0.014	−0.267 *
		BF_10_	0.170	0.511	1.949	0.717	0.158	29.587
	CARS11	Kendall’s tau	−0.025	0.010	0.084	−0.009	0.096	−0.038
		BF_10_	0.163	0.157	0.262	0.157	0.309	0.174
	CARS12	Kendall’s tau	−0.059	0.038	−0.093	−0.011	−0.047	−0.014
		BF_10_	0.202	0.174	0.296	0.157	0.183	0.158
	CARS13	Kendall’s tau	−0.049	0.143	−0.011	−0.085	0.084	0.001
		BF_10_	0.186	0.706	0.157	0.267	0.262	0.156
	CARS14	Kendall’s tau	−0.069	−0.003	−0.074	−0.038	−0.023	0.105
		BF_10_	0.222	0.156	0.232	0.173	0.162	0.350
	CARS15	Kendall’s tau	−0.018	0.166	−0.122	−0.096	0.032	0.004
		BF_10_	0.159	1.195	0.466	0.306	0.168	0.156
	Total CARS	Kendall’s tau	−0.047	−0.060	0.077	0.119	−0.033	−0.154
		BF_10_	0.183	0.203	0.241	0.440	0.169	0.897
Age < 14	CARS1	Kendall’s tau	−0.150	0.060	−0.108	0.057	−0.099	0.034
		BF_10_	0.571	0.222	0.334	0.218	0.304	0.196
	CARS2	Kendall’s tau	−0.170	−0.203	−0.092	0.154	−0.150	−0.006
		BF_10_	0.787	1.455	0.284	0.612	0.575	0.186
	CARS3	Kendall’s tau	0.126	0.050	0.076	−0.073	0.073	0.250
		BF_10_	0.410	0.210	0.248	0.242	0.243	4.233
	CARS4	Kendall’s tau	−0.137	0.132	−0.201	0.000	−0.067	−0.028
		BF_10_	0.473	0.444	1.401	0.185	0.233	0.193
	CARS5	Kendall’s tau	0.065	0.111	−0.085	−0.030	−0.083	0.076
		BF_10_	0.230	0.345	0.265	0.194	0.261	0.247
	CARS6	Kendall’s tau	−0.019	0.010	−0.061	0.012	−0.053	0.103
		BF_10_	0.189	0.186	0.224	0.187	0.213	0.316
	CARS7	Kendall’s tau	−0.224	0.034	0.156	0.189	−0.047	−0.264
		BF_10_	2.299	0.196	0.630	1.114	0.207	6.164
	CARS8	Kendall’s tau	−0.069	0.055	−0.032	0.055	−0.108	0.094
		BF_10_	0.235	0.215	0.195	0.216	0.333	0.288
	CARS9	Kendall’s tau	0.200	0.035	−0.254	−0.220	0.041	0.383 ***
		BF_10_	1.369	0.197	4.739	2.116	0.201	287.587
	CARS10	Kendall’s tau	−0.011	0.172	−0.200	−0.087	−0.016	0.116
		BF_10_	0.187	0.819	1.379	0.271	0.188	0.363
	CARS11	Kendall’s tau	0.063	0.057	−0.116	−0.037	−0.054	0.090
		BF_10_	0.226	0.218	0.363	0.199	0.215	0.278
	CARS12	Kendall’s tau	0.063	0.003	0.035	−0.032	0.096	0.047
		BF_10_	0.227	0.185	0.197	0.195	0.294	0.207
	CARS13	Kendall’s tau	−0.167	−0.072	−0.106	0.101	−0.148	−0.094
		BF_10_	0.757	0.241	0.325	0.310	0.557	0.290
	CARS14	Kendall’s tau	−0.011	−0.152	0.075	0.087	−0.014	−0.134
		BF_10_	0.186	0.596	0.245	0.272	0.187	0.456
	CARS15	Kendall’s tau	−0.198	0.062	−0.174	0.001	−0.068	0.169
		BF_10_	1.326	0.226	0.854	0.185	0.233	0.785
	Total CARS	Kendall’s tau	−0.092	0.006	−0.089	0.083	−0.131	0.053
		BF_10_	0.283	0.186	0.275	0.262	0.438	0.214

BF_10_, Bayes Factor in favor of H1 over H0 hypothesis; * BF_10_ > 10, ** BF_10_ > 30, *** BF_10_ > 100.

**Table 7:** Correlation between U-Mn, U-Cd, and biochemical variables with CARS scores in group of patients based on their sex.

Group	Variable	Statistic	U-Mn	U-Cd	fT3	fT4	fT3/fT4	TSH
Male	CARS1	Kendall’s tau	−0.067	−0.029	−0.003	0.104	−0.089	−0.037
		BF_10_	0.213	0.144	0.132	0.410	0.301	0.152
	CARS2	Kendall’s tau	−0.010	−0.125	−0.064	0.157	−0.218 *	0.040
		BF_10_	0.133	0.691	0.204	1.750	19.979	0.156
	CARS3	Kendall’s tau	0.083	0.180	−0.035	−0.085	0.046	0.062
		BF_10_	0.273	4.001	0.150	0.284	0.164	0.197
	CARS4	Kendall’s tau	−0.096	−0.059	0.028	0.138	−0.127	−0.132
		BF_10_	0.348	0.190	0.143	0.980	0.712	0.828
	CARS5	Kendall’s tau	0.114	0.073	−0.080	−0.070	−0.006	0.064
		BF_10_	0.521	0.230	0.260	0.221	0.132	0.203
	CARS6	Kendall’s tau	−0.005	−0.091	0.079	0.072	−0.045	−0.046
		BF_10_	0.132	0.315	0.255	0.229	0.163	0.165
	CARS7	Kendall’s tau	−0.088	−0.136	0.255 ***	0.265 ***	−0.092	−0.269 ***
		BF_10_	0.299	0.936	121.436	213.850	0.323	273.598
	CARS8	Kendall’s tau	−0.100	0.037	−0.009	0.046	−0.027	−0.015
		BF_10_	0.379	0.152	0.133	0.165	0.142	0.135
	CARS9	Kendall’s tau	0.141	0.073	−0.141	−0.106	0.006	0.190
		BF_10_	1.068	0.232	1.063	0.431	0.132	5.817
	CARS10	Kendall’s tau	−0.031	0.046	−0.042	5.175 × 10^−4^	−0.008	−0.053
		BF_10_	0.145	0.165	0.159	0.132	0.133	0.178
	CARS11	Kendall’s tau	0.065	0.031	−0.008	−0.044	0.035	0.028
		BF_10_	0.205	0.145	0.133	0.161	0.150	0.143
	CARS12	Kendall’s tau	0.036	0.041	−0.019	−0.052	0.045	0.018
		BF_10_	0.151	0.158	0.137	0.175	0.163	0.136
	CARS13	Kendall’s tau	−0.096	0.080	−0.056	−0.010	−0.006	−0.073
		BF_10_	0.346	0.257	0.183	0.133	0.132	0.230
	CARS14	Kendall’s tau	−0.032	−0.039	0.019	−0.029	0.054	−0.008
		BF_10_	0.147	0.154	0.137	0.144	0.180	0.133
	CARS15	Kendall’s tau	−0.069	0.097	−0.143	−0.032	−0.049	0.075
		BF_10_	0.217	0.355	1.136	0.147	0.169	0.238
	Total CARS	Kendall’s tau	−0.029	−0.031	0.001	0.090	−0.079	−0.069
		BF_10_	0.144	0.146	0.132	0.307	0.253	0.217
Female	CARS1	Kendall’s tau	−0.095	0.005	−0.168	0.134	−0.274	−0.147
		BF_10_	0.332	0.279	0.478	0.392	1.167	0.420
	CARS2	Kendall’s tau	0.067	0.083	−0.245	−0.214	−0.017	−0.023
		BF_10_	0.304	0.319	0.878	0.669	0.281	0.282
	CARS3	Kendall’s tau	0.019	−0.166	0.145	0.087	0.068	−0.190
		BF_10_	0.281	0.471	0.417	0.323	0.304	0.554
	CARS4	Kendall’s tau	−0.028	−0.077	0.040	0.090	−0.221	0.102
		BF_10_	0.283	0.313	0.288	0.325	0.706	0.341
	CARS5	Kendall’s tau	−0.328	0.149	0.047	−0.023	0.029	−0.053
		BF_10_	2.155	0.426	0.291	0.282	0.283	0.294
	CARS6	Kendall’s tau	−0.249	0.225	0.243	−0.006	0.158	−0.273
		BF_10_	0.907	0.732	0.858	0.279	0.448	1.146
	CARS7	Kendall’s tau	−0.262	−0.026	0.370	0.270	−0.026	−0.279
		BF_10_	1.030	0.283	3.742	1.115	0.283	1.230
	CARS8	Kendall’s tau	−0.233	−0.232	0.413	0.132	0.147	−0.228
		BF_10_	0.787	0.780	7.155	0.389	0.422	0.748
	CARS9	Kendall’s tau	0.000	−0.151	0.335	0.041	0.221	−0.120
		BF_10_	0.279	0.432	2.361	0.288	0.709	0.367
	CARS10	Kendall’s tau	−0.074	−0.129	0.549 **	0.280	0.037	−0.379
		BF_10_	0.310	0.383	83.041	1.244	0.286	4.294
	CARS11	Kendall’s tau	−0.106	−0.078	0.218	0.040	0.078	−0.149
		BF_10_	0.346	0.313	0.687	0.287	0.313	0.427
	CARS12	Kendall’s tau	−0.062	−0.294	0.090	0.262	−0.170	−0.223
		BF_10_	0.300	1.443	0.326	1.026	0.484	0.719
	CARS13	Kendall’s tau	−0.088	−0.071	0.022	−0.028	−0.071	0.079
		BF_10_	0.323	0.307	0.282	0.283	0.307	0.314
	CARS14	Kendall’s tau	−0.124	−0.091	−0.165	0.049	−0.236	0.044
		BF_10_	0.373	0.327	0.469	0.292	0.802	0.290
	CARS15	Kendall’s tau	−0.163	−0.063	0.172	0.085	0.005	−0.103
		BF_10_	0.464	0.301	0.491	0.320	0.279	0.341
	Total CARS	Kendall’s tau	−0.228	−0.014	0.178	0.132	−0.024	−0.134
		BF_10_	0.748	0.280	0.511	0.389	0.282	0.393

BF_10_, Bayes Factor in favor of H1 over H0 hypothesis; * BF_10_ > 10, ** BF_10_ > 30, *** BF_10_ > 100.

## Data Availability

The authors confirm that the data supporting the findings of this study are available within the article.

## References

[1] ICD-10 Version: 2016. apps.who.int.

[2] APA (2013). Autism spectrum disorder, 299.00 (F84.0). American Psychiatric Association Diagnostic and Statistical Manual of Mental Disorders.

[3] Weintraub K. (2011). The Prevalence Puzzle: Autism Counts. Nature.

[4] Elsabbagh M., Divan G., Koh Y.-J., Kim Y.S., Kauchali S., Marcín C., Montiel-Nava C., Patel V., Paula C.S., Wang C. (2012). Global Prevalence of Autism and Other Pervasive Developmental Disorders. Autism Res..

[5] Baxter A.J., Brugha T.S., Erskine H.E., Scheurer R.W., Vos T., Scott J.G. (2015). The Epidemiology and Global Burden of Autism Spectrum Disorders. Psychol. Med..

[6] Global Burden of Disease Collaborative Network (2017). Global Burden of Disease Study 2016 (GBD 2016) Cause-Specific Mortality 1980–2016.

[7] https://www.cdc.gov/ncbddd/autism/data.html.

[8] https://www.nik.gov.pl/aktualnosci/wsparcie-osob-z-autyzmem-i-zespolem-aspergera.html.

[9] Lavelle T.A., Weinstein M.C., Newhouse J.P., Munir K., Kuhlthau K.A., Prosser L.A. (2014). Economic Burden of Childhood Autism Spectrum Disorders. Pediatrics.

[10] Cakir J., Frye R.E., Walker S.J. (2020). The Lifetime Social Cost of Autism: 1990–2029. Res. Autism Spectr. Disord..

[11] Marshall C.R., Noor A., Vincent J.B., Lionel A.C., Feuk L., Skaug J., Shago M., Moessner R., Pinto D., Ren Y. (2008). Structural Variation of Chromosomes in Autism Spectrum Disorder. Am. J. Hum. Genet..

[12] Kim S.-J., Silva R.M., Flores C.G., Jacob S., Guter S., Valcante G., Zaytoun A.M., Cook E.H., Badner J.A. (2011). A Quantitative Association Study of SLC25A12 and Restricted Repetitive Behavior Traits in Autism Spectrum Disorders. Mol. Autism.

[13] Huguet G., Ey E., Bourgeron T. (2013). The Genetic Landscapes of Autism Spectrum Disorders. Annu. Rev. Genom. Hum. Genet..

[14] Park H.R., Lee J.M., Moon H.E., Lee D.S., Kim B.-N., Kim J., Kim D.G., Paek S.H. (2016). A Short Review on the Current Under-standing of Autism Spectrum Disorders. Exp. Neurobiol..

[15] Schaefer G.B., Mendelsohn N.J., Professional Practice and Guidelines Committee (2013). Clinical Genetics Evaluation in Identifying the Etiology of Autism Spectrum Disorders: 2013 Guideline Revisions. Genet. Med..

[16] Hawari I., Eskandar M.B., Alzeer S. (2020). The Role of Lead, Manganese, and Zinc in Autism Spectrum Disorders (ASDs) and Attention-Deficient Hyperactivity Disorder (ADHD): A Case-Control Study on Syrian Children Affected by the Syrian Crisis. Biol. Trace Elem. Res..

[17] Agency for Toxic Substances and Disease Registry. https://www.atsdr.cdc.gov/index.html.

[18] Soldin O.P., Aschner M. (2007). Effects of Manganese on Thyroid Hormone Homeostasis: Potential Links. Neurotoxicology.

[19] Harischandra D.S., Ghaisas S., Zenitsky G., Jin H., Kanthasamy A., Anantharam V., Kanthasamy A.G. (2019). Manganese-Induced Neurotoxicity: New Insights into the Triad of Protein Misfolding, Mitochondrial Impairment, and Neuroinflammation. Front. Neurosci..

[20] Rahbar M.H., Samms-Vaughan M., Ma J., Bressler J., Dickerson A.S., Hessabi M., Loveland K.A., Grove M.L., Shakespeare-Pellington S., Beecher C. (2015). Synergic Effect of GSTP1 and Blood Manganese Concentrations in Autism Spectrum Disorder. Res. Autism Spectr. Disord..

[21] Martinez-Finley E., Chakraborty S., Fretham S.J.B., Aschner M. (2012). Cellular transport and homeostasis of essential and nonessential metals. Metallomics.

[22] Jancic S.A., Stosic B.Z. (2014). Cadmium Effects on the Thyroid Gland. Vitam. Horm..

[23] Genchi G., Sinicropi M.S., Lauria G., Carocci A., Catalano A. (2020). The Effects of Cadmium Toxicity. Int. J. Environ. Res. Public Health.

[24] Shahid M.A., Ashraf M.A., Sharma S. (2021). Physiology, Thyroid Hormone. StatPearls.

[25] Uetani M., Kobayashi E., Suwazono Y., Honda R., Nishijo M., Nakagawa H., Kido T., Nogawa K. (2006). Tissue cadmium (Cd) concentrations of people living in a Cd polluted area, Japan. BioMetals.

[26] Godt J., Scheidig F., Grosse-Siestrup C., Esche V., Brandenburg P., Reich A., Groneberg D.A. (2006). The Toxicity of Cadmium and Resulting Hazards for Human Health. J. Occup. Med. Toxicol..

[27] Sherwin A.C., Flach F.F., Stokes P.E. (1958). Treatment of Psychoses in Early Childhood with Trilodothyronine. Am. J. Psychiatry.

[28] Hashimoto T., Aihara R., Tayama M., Miyazaki M., Shirakawa Y., Kuroda Y. (1991). Reduced Thyroid-Stimulating Hormone Response to Thyrotropin-Releasing Hormone in Autistic Male. Dev. Med. Child Neurol..

[29] Nir I., Meir D., Zilber N., Knobler H., Hadjez J., Lerner Y. (1995). Brief Report: Circadian Melatonin, Thyroid-Stimulating Hor-mone, Prolactin, and Cortisol Levels in Serum of Young Adults with Autism. J. Autism Dev. Disord..

[30] Gillberg I.C., Gillberg C., Kopp S. (1992). Hypothyroidism and Autism Spectrum Disorders. Child Psychol. Psychiatry Allied Discip..

[31] Hoshiko S., Grether J.K., Windham G.C., Smith D., Fessel K. (2011). Are Thyroid Hormone Concentrations at Birth Associated with Subsequent Autism Diagnosis?. Autism Res..

[32] Frye R.E., Sequeira J.M., Quadros E., Rossignol D. (2014). Folate Receptor Alpha Auto-antibodies Modulate Thyroid Function in Autism Spectrum Disorder. N. Am. J. Med. Sci..

[33] Molloy C.A., Morrow A.L., Meinzen-Derr J., Dawson G., Bernier R., Dunn M., Hyman S.L., McMahon W.M., Goudie-Nice J., Hepburn S. (2006). Familial Autoimmune Thyroid Disease as a Risk Factor for Regression in Children with Autism Spectrum Disorder: A CPEA Study. J. Autism Dev. Disord..

[34] Getahun D., Jacobsen S., Fassett M., Wing D.A., Xiang A.H., Chiu V.Y., Peltier M.R. (2018). Association between maternal hypothyroidism and autism spectrum disorders in children. Pediatr. Res..

[35] Cohen D.J., Young J.G., Lowe T.L., Harcherik D. (1980). Thyroid Hormone in Autistic Children. J. Autism Dev. Disord..

[36] Soldin O.P., Lai S., Lamm S.H., Mosee S. (2003). Lack of a Relation Between Human Neonatal Thyroxine and Pediatric Neurobe-havioral Disorders. Thyroid.

[37] Gorini F., Muratori F., Morales M.A. (2014). The Role of Heavy Metal Pollution in Neurobehavioral Disorders: A Focus on Autism. Rev. J. Autism Dev. Disord..

[38] Bondy S.C. (2010). The Neurotoxicity of Environmental Aluminum Is Still an Issue. Neurotoxicology.

[39] Branca J.J.V., Morucci G., Pacini A. (2018). Cadmium-Induced Neurotoxicity: Still Much Ado. Neural Regen. Res..

[40] Yasuda H., Yonashiro T., Yoshida K., Shibazaki T., Ishii T., Tsutsui T. (2005). High Toxic Metal Levels in Scalp Hair of Infants and Children. Biomed. Res. Trace Elem..

[41] Yasuda H., Yasuda Y., Tsutsui T. (2013). Estimation of autistic children by metallomics analysis. Sci. Rep..

[42] Rossignol D.A., Genuis S.J., Frye R.E. (2014). Environmental toxicants and autism spectrum disorders: A systematic review. Transl. Psychiatry.

[43] Skalny A.V., Simashkova N.V., Klyushnik T.P., Grabeklis A.R., Bjørklund G., Skalnaya M.G., Nikonorov A.A., Tinkov A.A. (2017). Hair toxic and essential trace elements in children with autism spectrum disorder. Metab. Brain Dis..

[44] Frye R.E., Wynne R., Rose S., Slattery J., Delhey L., Tippett M., Kahler S.G., Bennury S.C., Melnyk S., Sequeira J.M. (2017). Thyroid dysfunction in children with autism spectrum disorder is associated with folate receptor α autoimmune disorder. J. Neuroendocrinol..

[45] Skalny A.V., Simashkova N.V., Klyushnik T.P., Grabeklis A.R., Radysh I.V., Skalnaya M.G., Tinkov A.A. (2017). Analysis of Hair Trace Elements in Children with Autism Spectrum Disorders and Communication Disorders. Biol. Trace Elem. Res..

[46] Lord C., Luyster R.J., Gotham K., Guthrie W. (2012). Autism Diagnostic Observation Schedule, Second Edition (ADOS-2) Manual (Part II): Toddler Module.

[47] Chojnicka I., Ploski R. (2012). Polish version of the ADI-R (Autism Diagnostic Interview-Revised). Psychiatr. Pol..

[48] Schopler E., Van Bourgondien M.E., Wellman G.J., Love S.R. (2010). The Childhood Autism Rating Scale.

[49] Kułaga Z., Rózdzynska-Swiatkowska A., Grajda A., Gurzkowska B., Wojtyło M., Gózdz M., Swiader A., Litwin M. (2015). Wartości referencyjne wysokości, masy ciała i wskaźnika masy ciała dla oceny wzrastania i stanu od żywienia dzieci i młodzieży w wieku 3–18 lat, 2013, 11. Stand. Med. Pediatr..

[50] Błażewicz A., Makarewicz A., Korona-Głowniak I., Dolliver W.R., Kocjan R. (2016). Iodine in autism spectrum disorders. J. Trace Elem. Med. Biol..

[51] Prystupa A., Błażewicz A., Kiciński P., Sak J.J., Niedziałek J., Załuska W. (2016). Serum Concentrations of Selected Heavy Metals in Patients with Alcoholic Cirrhosis from the Lublin Region in Eastern Poland. Int. J. Environ. Res. Public Health..

[52] FT3 (Wolna Trijodotyronina)|Synevo. https://www.synevo.pl/ft3/.

[53] Önsesveren I., Barjaktarovic M., Chaker L., de Rijke Y.B., Jaddoe V.W.V., van Santen H.M., Visser T.J., Peeters R.P., Ko-revaar T.I.M. (2017). Childhood Thyroid Function Reference Ranges and Determinants: A Literature Overview and a Prospective Cohort Study. Thyroid.

[54] Fisher D.A., Nelson J.C., Carlton E.I., Wilcox R.B. (2000). Maturation of human hypothalamic-pituitary-thyroid function and control. Thyroid.

[55] Grissom M., Kreutzer J.S., DeLuca J., Caplan B. (2011). Childhood Autism Rating Scales. Encyclopedia of Clinical Neuropsychology.

[56] Tachimori H., Osada H., Kurita H. (2003). Childhood Autism Rating Scale—Tokyo Version for Screening Pervasive Developmental Disorders. Psychiatry Clin. Neurosci..

[57] Alabdali A., Al-Ayadhi L., El-Ansary A. (2014). A key role for an impaired detoxification mechanism in the etiology and severity of autism spectrum disorders. Behav. Brain Funct..

[58] Blaurock-Busch E., Amin O.R., Rabah T. (2011). Heavy metals and trace elements in hair and urine of a sample of Arab children with autistic spectrum disorder. Maedica.

[59] Adams J.B., Audhya T., McDonough-Means S., Rubin R.A., Quig D., Geis E., Gehn E., Loresto M., Mitchell J., Atwood S. (2013). Toxicological status of children with autism vs. neurotypical children and the association with autism severity. Biol. Trace Elem. Res..

[60] Williams M., Todd G., Roney N., Crawford J., Coles C., McClure P., Garey J., Zaccaria K., Citra M. (2012). Toxicological Profile for Manganese.

[61] Kaya Akyüzlü D., Kayaalti Z., Söylemez Y., Soylemezoglu T. (2014). Association between Autism and Arsenic, Lead, Cadmium, Manganese Levels in Hair and Urine. J. Pharm. Pharmacol..

[62] Chaumont A., Voisin C., Deumer G., Haufroid V., Annesi-Maesano I., Roels H., Thijs L., Staessen J., Bernard A. (2013). Associations of urinary cadmium with age and urinary proteins: Further evidence of physiological variations unrelated to metal accumulation and toxicity. Environ. Health Perspect..

[63] Skogheim T.S., Weyde K.V.F., Engel S.M., Aase H., Surén P., Øie M.G., Biele G., Reichborn-Kjennerud T., Caspersen I.H., Hornig M. (2021). Metal and Essential Element Concentrations during Pregnancy and Associations with Autism Spectrum Disorder and Attention-Deficit/Hyperactivity Disorder in Children. Environ. Int..

[64] Liu C., Hutchens S., Jursa T., Shawlot W., Polishchuk E.V., Polishchuk R.S., Dray B.K., Gore A.C., Aschner M., Smith D.R. (2017). Hypothyroidism Induced by Loss of the Manganese Efflux Transporter SLC30A10 May Be Explained by Reduced Thyroxine Production. J. Biol. Chem..

[65] Fiore M., Barone R., Copat C., Grasso A., Cristaldi A., Rizzo R., Ferrante M. (2020). Metal and Essential Element Levels in Hair and Association with Autism Severity. J. Trace Elem. Med. Biol..

[66] Kaur K., Suneja B., Jodhka S., Kaur J., Singh A., Singh S.R. (2021). Metallic Insignia in Primary Teeth: A Biomarker for Autism Spectrum Disorders. J. Indian Soc. Pedod. Prev. Dent..

[67] Favorito R., Chiarelli G., Grimaldi M.C., De Bonis S., Lancieri M., Ferrandino I. (2011). Bioaccumulation of Cadmium and Its Cytotoxic Effect on Zebrafish Brain. Chem. Ecol..

[68] Chen A., Kim S.S., Chung E., Dietrich K.N. (2013). Thyroid Hormones in Relation to Lead, Mercury, and Cadmium Exposure in the National Health and Nutrition Examination Survey, 2007–2008. Environ. Health Perspect..

[69] Christensen K.L.Y. (2013). Metals in Blood and Urine, and Thyroid Function among Adults in the United States 2007–2008. Int. J. Hyg. Environ. Health.

[70] Satarug S. (2018). Dietary Cadmium Intake and Its Effects on Kidneys. Toxics.

[71] Jain R.B., Choi Y.S. (2016). Interacting Effects of Selected Trace and Toxic Metals on Thyroid Function. Int. J. Environ. Health Res..

[72] Faroon O., Ashizawa A., Wright S., Tucker P., Jenkins K., Ingerman L., Rudisill C. (2012). Toxicological Profile for Cadmium.

[73] Buha A., Matovic V., Antonijevic B., Bulat Z., Curcic M., Renieri E.A., Tsatsakis A.M., Schweitzer A., Wallace D. (2018). Overview of Cadmium Thyroid Disrupting Effects and Mechanisms. Int. J. Mol. Sci..

[74] Marco E.J., Hinkley L.B., Hill S.S., Nagarajan S.S. (2011). Sensory processing in autism: A review of neurophysiologic findings. Pediatr. Res..

[75] Avallone B., Crispino R., Cerciello R., Simoniello P., Panzuto R., Motta C.M. (2015). Cadmium effects on the retina of adult Danio rerio. Comptes Rendus Biol..

[76] Wills N.K., Ramanujam V.M., Chang J., Kalariya N., Lewis J.R., Weng T.X., van Kuijk F.J. (2008). Cadmium accumulation in the human retina: Effects of age, gender, and cellular toxicity. Exp. Eye Res..

[77] Pamphlett R., Cherepanoff S., Too L.K., Kum Jew S., Doble P.A., Bishop D.P. (2020). The distribution of toxic metals in the human retina and optic nerve head: Implications for age-related macular degeneration. PLoS ONE.

[78] Green A.J., Hoyo C., Mattingly C.J., Luo Y., Tzeng J.-Y., Murphy S.K., Buchwalter D.B., Planchart A. (2018). Cadmium Exposure Increases the Risk of Juvenile Obesity: A Human and Zebrafish Comparative Study. Int. J. Obes..

[79] Filis P., Hombach-Klonisch S., Ayotte P., Nagrath N., Soffientini U., Klonisch T., O’Shaughnessy P., Fowler P.A. (2018). Maternal Smoking and High BMI Disrupt Thyroid Gland Development. BMC Med..

[80] Chung S.M., Moon J.S., Yoon J.S., Won K.C., Lee H.W. (2019). Sex-Specific Effects of Blood Cadmium on Thyroid Hormones and Thyroid Function Status: Korean Nationwide Cross-Sectional Study. J. Trace Elem. Med. Biol..

